# Self‐Assembling Peptide‐Adjuvant Conjugate (SaPAC) Platform for Precision Cancer Immunotherapy

**DOI:** 10.1002/advs.77632

**Published:** 2026-10-02

**Authors:** Yang‐Fan Wu, Jing‐Chu Hu, Ye‐Fan Hu, Wen‐Jun Li, Li Rong, Ren‐Hao Li, Yuan Yao, Lehan Hu, Xiao‐Lei Wang, Bao‐Zhong Zhang, Yicheng Lu, Shing‐Fung Chow, Canhui Su, Thomas Yau, Clive Yik‐Sham Chung, Jian‐Dong Huang

**Affiliations:** ^1^ School of Biomedical Sciences and School of Biomedical Engineering Li Ka Shing Faculty of Medicine The University of Hong Kong Pokfulam Hong Kong SAR People's Republic of China; ^2^ Centre of Cancer Medicine School of Clinical Medicine The University of Hong Kong Pokfulam Hong Kong People's Republic of China; ^3^ BayVax Biotech Limited Hong Kong Science Park Pak Shek Kok New Territories Hong Kong People's Republic of China; ^4^ Shenzhen BayVax Biotech Limited Shenzhen People's Republic of China; ^5^ Key Laboratory of Quantitative Synthetic Biology, Shenzhen Institute of Synthetic Biology Shenzhen Institutes of Advanced Technology, Chinese Academy of Sciences Shenzhen People's Republic of China; ^6^ Department of Pharmacology and Pharmacy Li Ka Shing Faculty of Medicine, The University of Hong Kong Pokfulam Hong Kong People's Republic of China; ^7^ Department of Pathology School of Clinical Medicine Li Ka Shing Faculty of Medicine The University of Hong Kong Hong Kong People's Republic of China; ^8^ Centre for Virology, Vaccinology and Therapeutics Hong Kong Science and Technology Park Hong Kong Special Administrative Region People's Republic of China; ^9^ Shenzhen Key Laboratory for Cancer Metastasis and Personalized Therapy Department of Clinical Oncology The University of Hong Kong‐Shenzhen Hospital Shenzhen Guangdong Province People's Republic of China; ^10^ Materials Innovation Institute for Life Sciences and Energy (MILES) HKU‐SIRI Shenzhen People's Republic of China

**Keywords:** lymph node delivery, peptide‐adjuvant conjugate, precision cancer immunotherapy, self‐assembled peptide nanovaccine, TLR7 conjugate neoantigen vaccine

## Abstract

Peptide cancer vaccines often suffer from poor lymphatic drainage, limited antigen‐presenting cell uptake, and weak innate immune activation. To overcome these barriers, we engineered the Self‐Assembling Peptide‐Adjuvant Conjugate (SaPAC) platform, which covalently links neoantigen epitopes to the TLR7 agonist 1V209 through site‐selective lysine conjugation. Synthesized by Fmoc solid‐phase peptide synthesis, SaPAC conjugates self‐assemble into cationic nanoparticles with hydrodynamic diameters of approximately 100–200 nm. Compared with unconjugated self‐assembling peptide nanoparticles mixed with soluble adjuvant, SaPAC nanoparticles enhanced lymphatic drainage and antigen persistence in draining lymph nodes. Mechanistically, SaPAC activated the TLR7‐MyD88 axis, promoted plasmacytoid dendritic cell and macrophage recruitment, drove APC maturation (CD80^+^CD86^+^), and enhanced MHC‐I cross‐presentation to amplify antigen‐specific CD8+ T‐cell priming. Therapeutically, SaPAC monotherapy suppressed tumor growth in B16‐OVA melanoma and MB49 bladder carcinoma models by increasing intratumoral infiltration of activated CD8+ T cells and NK cells. In an orthotopic 4T1 triple‐negative breast cancer model, multivalent SaPAC vaccination synergized with anti‐PD‐1 blockade and achieved durable tumor suppression comparable to the Poly(I:C)‐adjuvanted benchmark without detectable systemic toxicity. Collectively, SaPAC represents a chemically defined platform that bridges innate and adaptive anti‐tumor immunity.

## Graphical abstract

A self‐assembling peptide–adjuvant conjugate links neoantigens to TLR7 agonism, forming cationic nanoparticles that enhance lymph‐node retention, dendritic‐cell activation, and antitumor T‐cell immunity. This chemically defined platform shows how synchronized antigen and innate stimulation can be adapted across melanoma, bladder cancer, and triple‐negative breast cancer models.

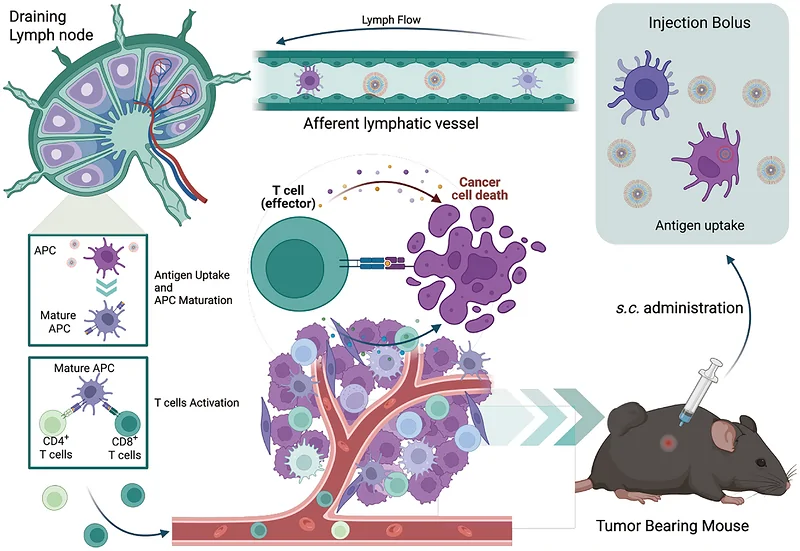

## 1 Introduction

Peptides are attractive biopharmaceuticals because of their target specificity, favorable safety profiles, modest production costs, and rapid manufacturability [1, 2]. However, unmodified peptides often show poor aqueous solubility, rapid proteolytic degradation, and short in vivo half‐lives, limiting their utility as vaccines and therapeutics [3, 4]. Chemical modification can improve peptide stability and pharmacokinetics, as illustrated by semaglutide [5, 6]. For peptide cancer vaccines, analogous engineering strategies remain needed to couple antigen delivery with innate immune activation.

Cancer neoantigen vaccines exploit tumor‐specific somatic mutations that generate peptides presented by major histocompatibility complex (MHC) molecules [7, 8]. Because neoantigens are absent from healthy tissues, they can elicit tumor‐restricted T‐cell responses with reduced risk of autoimmunity. Recent clinical studies across melanoma, lung cancer, glioblastoma, hepatocellular carcinoma, and mixed solid tumors show that personalized peptide vaccines can induce neoantigen‐specific CD8+ and CD4+ T‐cell responses, particularly when combined with checkpoint blockade [9, 10, 11, 12, 13, 14]. These advances also emphasize the need for delivery systems and adjuvant strategies that improve vaccine potency and breadth.

Peptide‐based neoantigen vaccines face linked delivery and immunogenicity barriers: poor solubility, inefficient trafficking to draining lymph nodes (LNs), limited uptake and processing by antigen‐presenting cells (APCs), and weak innate immune activation [8, 15]. Post‐modification strategies can tune hydrophobicity and promote self‐assembly into nanostructures that enhance antigen retention, lymphatic drainage, and APC access [16, 17, 18, 19, 20, 21]. Prior peptide–TLR ligand conjugate and self‐assembling peptide‐adjuvant vaccine studies support the concept that covalent co‐delivery of antigen and adjuvant can improve CD8+ T‐cell immunity relative to simple mixtures [18, 22, 23, 24].

To address these challenges, we developed the Self‐Assembling Peptide‐Adjuvant Conjugate (SaPAC) platform. SaPN denotes the antigen‐bearing **S**elf‐**a**ssembling **P**eptide **N**anoparticle scaffold without covalently attached 1V209, whereas SaPAC denotes the corresponding peptide‐1V209 conjugate nanoparticle. In physical‐mixture controls, 1V209 was administered in unconjugated form. This modular design enables hydrophobicity and charge tuning, generates cationic nanoparticles of approximately 100–200 nm under hydrated conditions, enhances LN bioavailability and dendritic cell (D) uptake, and synchronizes innate TLR7 stimulation with adaptive antigen presentation.

## 2 Results

### 2.1 The SaPN Precursor Nanoparticle Scaffold Establishes an Antigen Depot and Favorable Systemic Pharmacokinetics

According to [25], parenterally administered antigens typically follow one of four physiological fates: systemic vascular circulation, tissue‐level degradation, trafficking to secondary lymphatic organs, or capture by resident antigen‐presenting cells (APCs) (Figure S1). We hypothesized that our self‐assembling peptide nanoparticle (SaPN) platform would modulate these dynamics by slowing neoantigen release into systemic circulation and protecting the peptide cargo from premature proteolytic clearance within the interstitial tissue. We therefore first assessed whether the adjuvant‐free SaPN precursor could improve antigen disposition before examining the 1V209‐conjugated SaPAC platform.

SaPN denotes the antigen‐bearing precursor scaffold of the SaPAC platform. In the model (Figure 1A), SaPN‐OVA comprises an N‐terminal lysine‐rich cationic region, cathepsin‐sensitive SLVR linkers [18, 26], and the MHC‐I‐restricted SIINFEKL epitope (OVA_257‐264_, herein denoted as OVA) derived from ovalbumin [27], but contains no covalently attached adjuvant. 1V209 is a small‐molecule Toll‐like receptor 7 (TLR7) agonist bearing a carboxylic acid group that enables site‐selective amide coupling to lysine side chains. Site‐selective conjugation of 1V209 to designated lysines in the SaPN precursor sequence generates SaPAC‐OVA, thereby integrating antigen delivery and TLR7 stimulation within one self‐assembling construct. In control formulations, SaPN‐OVA + 1V209 and OVA + 1V209 denote physical mixtures with unconjugated 1V209, while Poly(I:C)‐adjuvanted vaccines serve as a mechanistically distinct benchmark.

The ability of these constructs to self‐assemble arises from the noncovalent interactions encoded by their molecular architecture. Peptide assembly can involve hydrophobic association, hydrogen bonding, electrostatic interactions, and *π*–*π* stacking [4, 28], and charge‐modified peptide–TLR7/8 agonist conjugates provide a closely related precedent for chemically programmed nanoparticle formation [18]. Accordingly, SaPN‐OVA and SaPAC‐OVA are proposed to assemble through an amphiphilic balance between their sequence elements (Figure 1A and Figure S3B): the N‐terminal lysine‐rich segment provides a cationic, hydrophilic domain; nonpolar residues within the SLVR linkers and antigen‐containing region contribute hydrophobic association; and, in SaPAC, the aromatic and nonpolar portions of conjugated 1V209 may further strengthen this association. Alanine‐ or tryptophan‐containing C‐terminal spacer residues tune the balance among variants. Hydrophobic association promotes intermolecular assembly, whereas hydration and electrostatic repulsion of the lysine‐rich region stabilize the resulting cationic nanoparticles in aqueous media.

**FIGURE 1:**
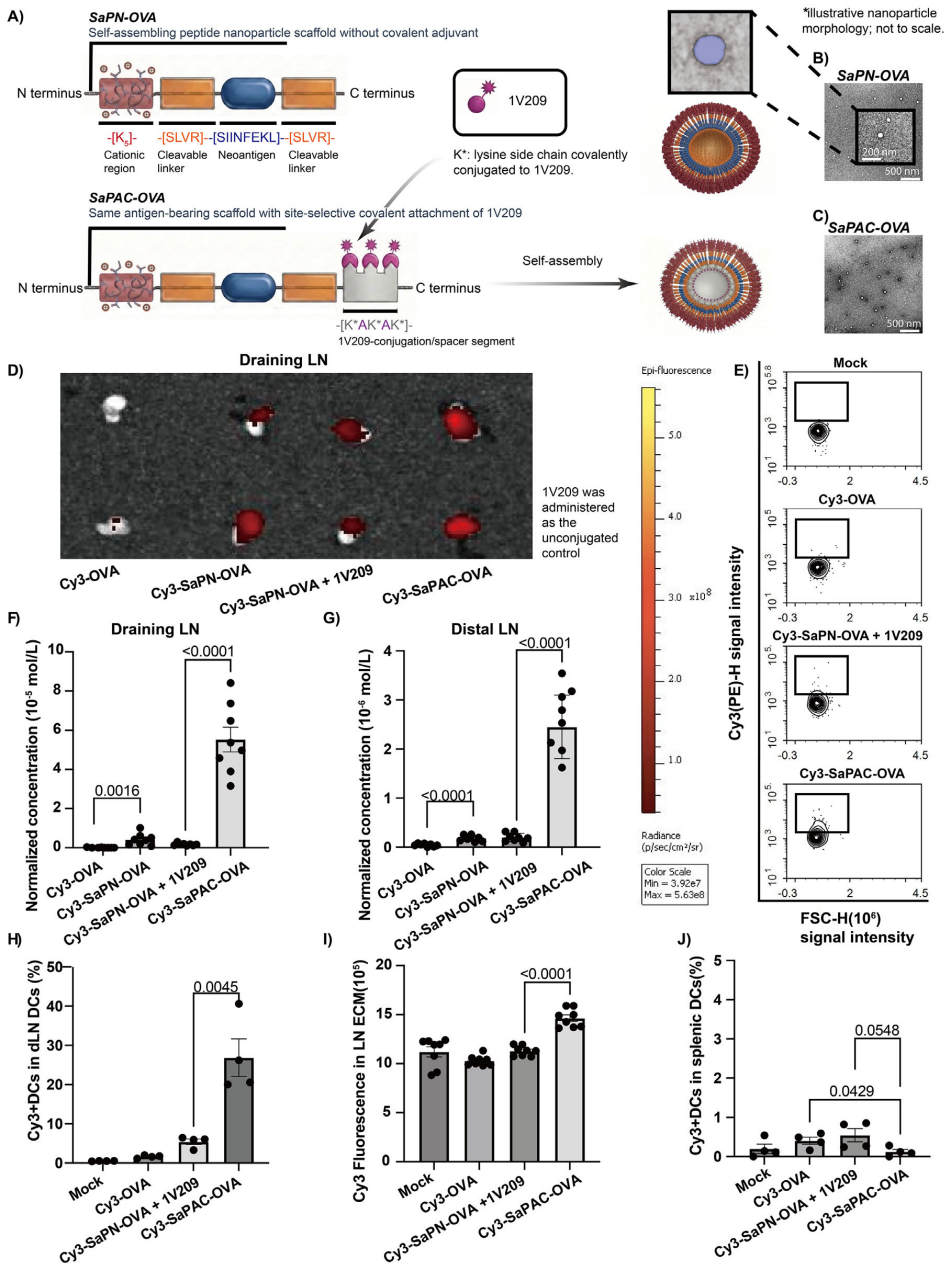
SaPAC nanoparticles sustain neoantigen bioavailability in lymph nodes and enhance dendritic cell uptake. (A) Design of SaPN‐OVA and SaPAC‐OVA. SaPN‐OVA comprises a cationic region, enzyme‐cleavable linkers, and the OVA epitope without a covalent adjuvant. 1V209 is a Toll‐like receptor 7 (TLR7) agonist. SaPAC‐OVA is generated by site‐selective conjugation of the TLR7 agonist 1V209 to designated lysine side chains (K^*^); K^*^ denotes a lysine side chain covalently conjugated to 1V209. OVA denotes the SIINFEKL peptide epitope derived from ovalbumin. Representative TEM images of (B) SaPN‐OVA and (C) SaPAC‐OVA nanoparticles. (D) Cy3‐labeled peptides or vaccine nanoparticles were injected subcutaneously; draining LNs and organs were excised 24 h later and analyzed by IVIS fluorescence imaging. (E) Draining LNs were dissociated for flow cytometric analysis of DCs. (F, G) Normalized Cy3 fluorescence in draining and distal LNs. (H) Frequency of Cy3+ DCs. (I) Supernatant fluorescence after LN dissociation. (J) Frequency of Cy3+ splenic DCs. Data are mean ± SEM; significance was assessed by unpaired two‐tailed Student's t‐test (^*^ *p* < 0.05, ^**^ *p* < 0.01, ^***^ *p* < 0.001).

Following subcutaneous injection, antigen concentration in the blood is governed by the equilibrium between the systemic absorption rate (*K_abs_*) and the systemic clearance rate (*K_clear_*) (Figure S1E). These kinetic rates dictate the effective bioavailability of the antigen within the draining lymph nodes (dLNs) and distal lymphoid tissues (Figure S1B). Specifically, a reduced *K_abs_* favors increased partitioning and flux into the dLNs, whereas a reduced *K_clear_* extends the circulating half‐life, providing prolonged exposure to distal sites.

To quantify these blood dynamics, we first confirmed that FITC‐OVA and FITC‐SaPN‐OVA had comparable fluorescence calibration curves (EC50, *p* = 0.7803; Figure S2C), minimizing bias from construct‐dependent fluorescence intensity. The formulations were then administered intravenously or subcutaneously, and serum fluorescence curves were fitted using the pharmacokinetic equations in Figure S1E, with datasets shown in Figures S1C,D and S2D. The fitted parameters showed lower systemic absorption and clearance rates for FITC‐SaPN‐OVA than for FITC‐OVA (Figure S1F,G), indicating slower entry into the circulation and prolonged systemic persistence.

Consistent with its slower systemic absorption, FITC‐SaPN‐OVA produced stronger fluorescence in draining inguinal LNs than FITC‐OVA 6 h after subcutaneous administration (*p* < 0.005; Figure S2B,E). Whole‐body IVIS imaging also showed prolonged retention of FITC‐SaPN‐OVA near the injection site, whereas FITC‐OVA dissipated rapidly (Figure S2A). Collectively, these findings indicate that SaPN‐OVA limits rapid vascular entry, prolongs local antigen availability, and improves delivery to dLNs.

### 2.2 Design and Characterization of SaPAC Self‐Assembling Peptide‐Adjuvant Nanoparticles for Neoantigen Delivery

Having established the pharmacokinetic behavior of the SaPN precursor, we next examined the synthesis and physicochemical properties of the 1V209‐conjugated SaPAC constructs. Figure 1A and Figure S3B map their shared architecture, including the N‐terminal cationic region, enzyme‐cleavable linkers, antigen epitope, C‐terminal conjugation/spacer segment, and designated 1V209‐conjugated lysines (K^*^), and distinguish SaPN‐OVA from SaPAC‐OVA and its sequence variants.

SaPAC peptide‐adjuvant conjugates were synthesized by Fmoc‐based solid‐phase peptide synthesis on Rink amide resin (Figure S3A). Orthogonally protected lysines enabled site‐selective 1V209 installation: Dde‐protected target lysines were selectively deprotected with 2% hydrazine in N,N‐dimethylformamide, while Boc‐protected non‐target lysines remained protected. The carboxylic acid of 1V209 was then coupled to exposed epsilon‐amino groups to form amide‐linked peptide‐adjuvant conjugates. The corresponding construct map in Figure S3B identifies the sequence‐level differences among SaPN‐OVA, SaPAC‐OVA, SaPAC(ii)‐OVA, and SaPAC(iii)‐OVA.

After global deprotection and resin cleavage, crude products were purified by reversed‐phase HPLC to >95% purity and confirmed by high‐resolution mass spectrometry. Figure S4A shows the RP‐HPLC profiles of SaPAC‐OVA and its variants; Figure S4B provides HR‐MS confirmation; Figure S4C,D show representative NTA size distributions for SaPN‐OVA and SaPAC‐OVA, and Figure S4E summarizes hydrodynamic diameter and zeta‐potential measurements across constructs. TEM, NTA/ZetaView, and zeta‐potential measurements characterize complementary aspects of morphology, hydrated particle size, and surface charge: TEM reports dry‐state primary structures, whereas NTA/ZetaView measures hydrodynamic diameters in buffer.

### 2.3 SaPAC Nanoparticles Sustain Neoantigen Bioavailability in Lymph Nodes and Enhance Dendritic Cell Uptake

To determine whether SaPAC nanoparticles sustained neoantigen bioavailability in lymph nodes (LNs), we tracked fluorescently labeled formulations after subcutaneous administration. Cy3‐SaPAC‐OVA, Cy3‐SaPN‐OVA, and Cy3‐OVA were compared. At 24 h post‐injection, draining inguinal LNs and major organs were excised for IVIS epifluorescence imaging (Figure 1D). Cy3 signals were normalized to construct‐specific EC50 values (Figure S5). Biodistribution analysis showed selective accumulation of Cy3‐SaPAC‐OVA in draining and distal LNs relative to other formulations (Figure 1F,G and Figure S6), indicating prolonged antigen retention in lymphoid tissues.

Draining LNs were then dissociated into supernatants and single‐cell suspensions (Figure 1E,I). Supernatant fluorescence measured by GE Amersham Typhoon 5 imaging was highest in the Cy3‐SaPAC‐OVA group (Figure 1I), supporting enhanced lymphatic delivery. Flow cytometry of LN and splenic DCs (CD45^+^CD3^−^CD19^−^MHCII^+^CD11c^+^) further showed the highest intracellular Cy3 signal in LN DCs from Cy3‐SaPAC‐OVA‐treated mice (Figure 1E,H,J). These results indicate that SaPAC‐OVA nanoparticles prolong antigen availability through both passive lymphatic drainage and DC‐associated transport.

### 2.4 SaPAC Drives Robust Dendritic Cell Cross‐Presentation and Acute T Cell Activation

Having validated that SaPAC enhanced neoantigen bioavailability in draining lymph nodes, we next examined whether the increased exposure to vaccines promoted DC maturation and subsequent T‐cell priming. To investigate release and DC‐mediated presentation of the OVA epitope (OVA) from SaPAC‐OVA, we first used the immortalized murine DC line DC2.4 (Figure S7). Antigen was matched at 8.75 × 10^−9^ mol per condition as OVA, 8.75 × 10^−9^ mol of SaPN‐OVA, 8.75 × 10^−9^ mol of SaPN‐OVA + 2.625 × 10^−8^ mol of 1V209, or 8.75 × 10^−9^ mol of SaPAC‐OVA. Because SaPAC‐OVA contains three covalently linked 1V209 moieties per construct, the SaPN‐OVA + 1V209 group received 2.625 × 10^−8^ mol of unconjugated 1V209, corresponding to three molar equivalents per antigen molecule. After 24 h, cells were analyzed by flow cytometry using antibodies specific for the OVA‐H2Kb complex. As shown in Figure S7, approximately 90% of DC2.4 cells presented the OVA epitope following co‐culture with SaPAC‐OVA, representing a nearly 10‐fold increase relative to SaPN‐OVA or SaPN‐OVA + 1V209. These findings suggest that SaPAC‐OVA facilitates sustained and robust antigen presentation. However, DC2.4 is a proliferative cell line with constitutively high expression of costimulatory molecules, which may confound assessments of DC maturation and surface‐biomarker profiles.

To address these constraints, bone marrow‐derived DCs (BMDCs) were generated from tibial marrow isolates and differentiated for 7 days in the presence of IL‐4 and GM‐CSF [29]. The resulting BMDCs were co‐cultured with the peptide or vaccine formulations, harvested, and subjected to flow cytometric analysis (Figure 2A–C). Notably, within the CD11c^+^MHCII^+^ subset, over 75% of cells from the SaPAC‐OVA group displayed OVA presentation via MHC class I (MHC‐I) molecules (Figure 2A), significantly surpassing the <50% observed with SaPN‐OVA or SaPN‐OVA + 1V209 treatments. These data indicate that SaPAC‐OVA potently drives cross‐presentation in DCs in vitro. We further evaluated costimulatory markers CD80 and CD86, which are essential for T cell priming. More than 20% of OVA‐H2K^b^^+^ BMDCs treated with SaPAC‐OVA co‐expressed CD80 and CD86 (Figure 2B,C), approximately 4‐fold higher than in the SaPN‐OVA + 1V209 group (Figure 2B,C), thereby demonstrating that SaPAC‐OVA induces profound DC maturation in vitro. While the SaPN‐OVA nanoparticle platform enhanced OVA uptake and presentation by DCs, it exhibited limited capacity to induce maturation, even when co‐administered with 1V209. In contrast, covalent conjugation to form SaPAC‐OVA elicited superior antigen presentation and DC maturation in vitro.

**FIGURE 2:**
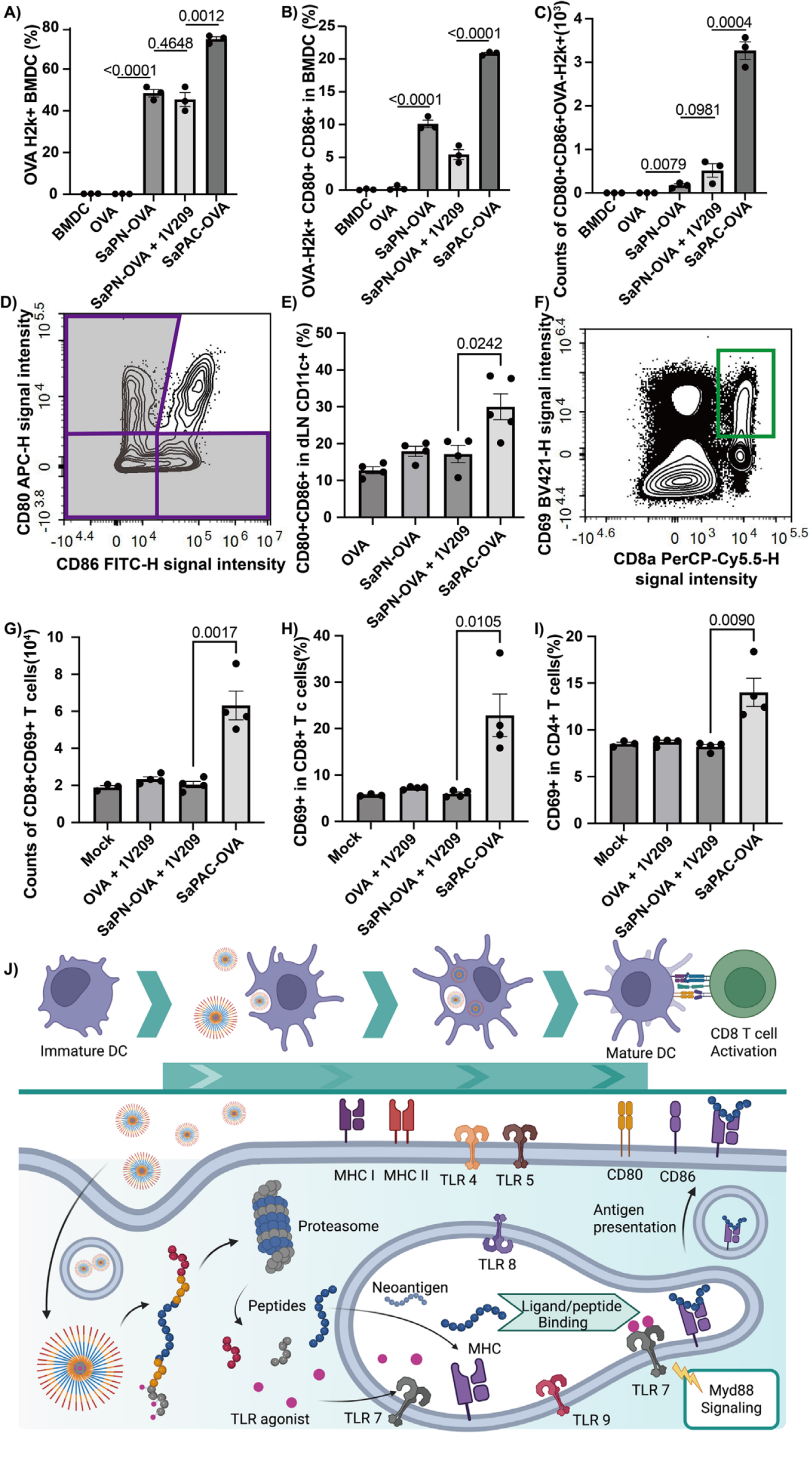
SaPAC drives robust dendritic cell cross‐presentation and acute T cell activation. (A–C) In vitro assessment of bone marrow‐derived DCs (BMDCs) treated with OVA epitope (OVA) or vaccine formulations: (A) OVA antigen presentation quantified by flow cytometry (anti‐SIINFEKL‐H‐2K^b^ tetramer); (B) frequency of mature DCs (CD80^+^CD86^+^ subpopulation); (C) absolute counts of mature DCs. (D) Representative flow cytometric gating strategy for CD11c^+^ DCs (pre‐gated as CD3^−^CD19^−^CD45^+^CD11c^+^) in dLNs. (E) Frequency of mature DCs (CD80^+^CD86^+^) in dLNs post‐vaccination (*n* = 4–6 mice/group; two‐tailed Student's t‐test). (F) Gating strategy for T cells (pre‐gated as CD3^+^CD45^+^) in dLNs. (G) Absolute counts of activated CD8^+^ T cells (CD69^+^). (H) Expression levels (MFI) of CD69 on CD8^+^ T cells. (I) Expression levels (MFI) of CD69 on CD4^+^ T cells. Data represent mean ± SEM; ^*^ *p* < 0.05, ^**^ *p* < 0.01, ^***^ *p* < 0.001 vs. control. (J) Schematic of DC maturation induced by SaPAC‐OVA. The design of SaPAC‐OVA enables co‐delivery of neoantigen (OVA epitope) and a TLR7 agonist efficiently internalized by DCs via endocytosis or phagocytosis. Inside DCs, the conjugate traffics from endosomes to the cytosol through ER‐phagosome fusion and ER‐associated degradation. Proteasomal cleavage at enzyme‐sensitive linkers releases OVA peptides for MHC I presentation, while the dissociated TLR7 agonist engages endosomal TLR7, triggering MyD88‐dependent signaling. This coordinated antigen processing and innate activation promotes DC maturation and enhanced T cell priming.

To extend these observations in vivo, mice were subcutaneously immunized with 2.5 × 10^−8^ mol antigen per group as SaPAC‐OVA, OVA, SaPN‐OVA, or SaPN‐OVA + 1V209 (Figure 2D–I). SaPAC‐OVA contains three covalently linked 1V209 moieties per construct; accordingly, the SaPN‐OVA + 1V209 group received 7.5 × 10^−8^ mol unconjugated 1V209, corresponding to three molar equivalents per antigen molecule. Draining LNs were excised 24 h post‐immunization and analyzed by flow cytometry. SaPAC‐OVA produced the highest frequency of CD80^+^CD86^+^ mature DCs among CD11c^+^ myeloid cells (Figure 2D,E). A second cohort was used to assess T‐cell activation (Figure 2F–H). SaPAC‐OVA‐driven DC maturation translated into increased numbers and CD69 expression of activated CD8^+^ T cells in dLNs, together with enhanced CD4^+^ T‐cell paracrine activation (Figure 2G–I).

Integrating the findings from Figures 1 and 2, the intracellular trafficking and processing pathway of SaPAC‐OVA within LNs is schematically illustrated in Figure 2J. The SaPAC‐OVA architecture ensures the synchronized delivery of the neoantigen (OVA epitope) and the TLR7 agonist to the LNs, followed by efficient internalization by DCs via endocytic or phagocytic pathways. Once internalized, a portion of the conjugate undergoes endosomal escape into the cytosol. Within the cytosolic compartment, proteasomal cleavage at the engineered enzyme‐sensitive linkers liberates the OVA epitope peptides for MHC‐I cross‐presentation. Concurrently, the TLR7 agonist engages endosomal TLR7, initiating the signaling cascades required for DC phenotypic maturation. This spatial‐temporal coordination of antigen processing and innate activation promotes robust DC maturation, ultimately driving potent and enhanced T cell priming.

### 2.5 SaPAC Enhances Neoantigen Immunogenicity Through Covalent Adjuvant Tethering and Balanced Cytokine Signaling

To test whether enhanced neoantigen immunogenicity was a general feature of peptide‐adjuvant nanoparticles, we synthesized SaPAC‐OVA variants with different C‐terminal residues (Figure 3A and Figure S3B). Amino acid composition was varied to tune hydrophobicity and amphiphilicity during SPPS [30]. Reversed‐phase HPLC profiles in Figure S4A confirmed the expected differences in retention, with SaPAC‐OVA showing the longest retention time (11.56 min), followed by SaPAC(iii)‐OVA (11.12 min) and SaPAC(ii)‐OVA (8.98 min). The shorter retention time of SaPAC(ii)‐OVA is consistent with relatively increased hydrophilicity.

**FIGURE 3:**
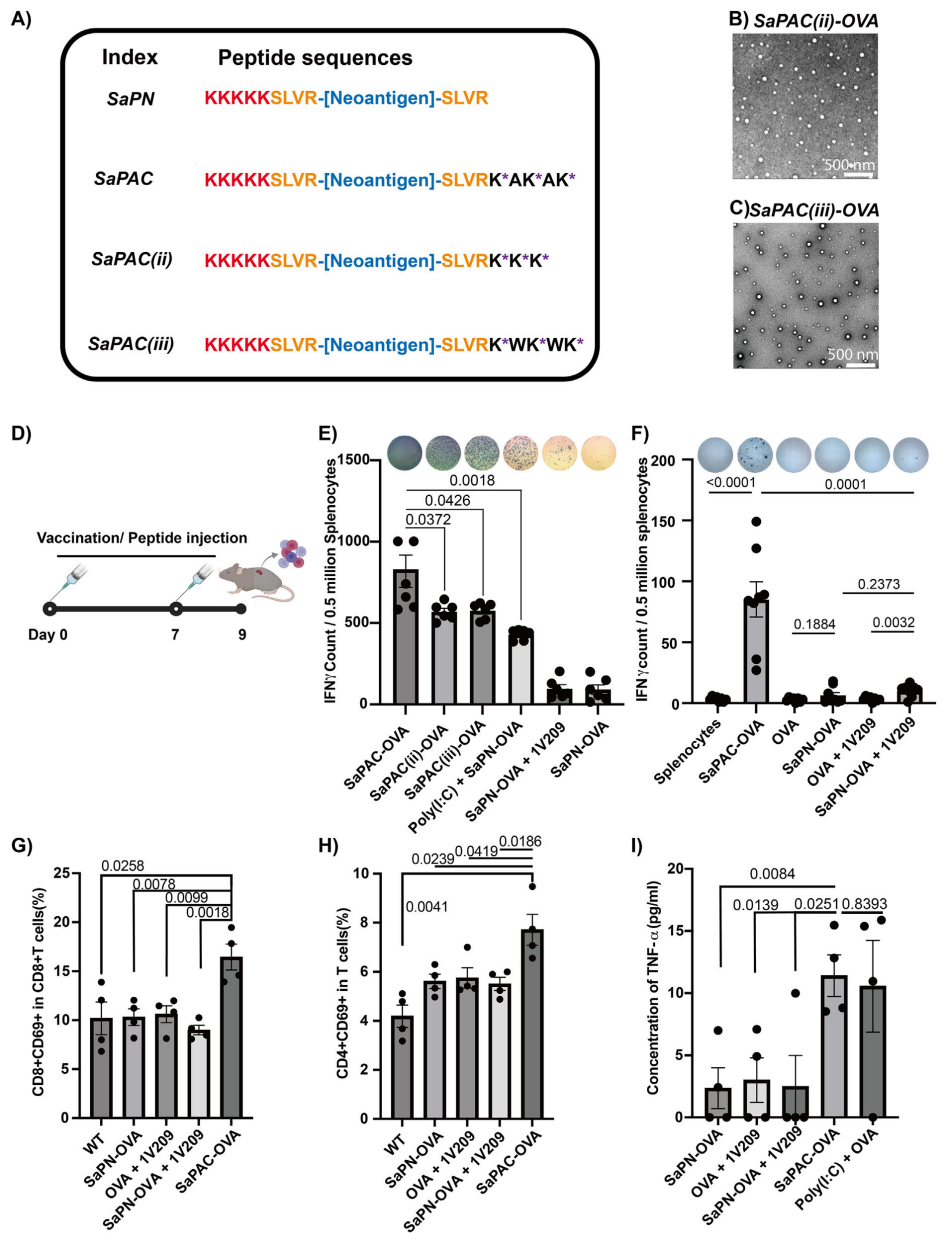
SaPAC elicits potent T‐cell activation and a balanced inflammatory cytokine profile. (A) Amino acid sequences of synthetic peptides and peptide‐adjuvant conjugates; K^*^ indicates lysine conjugated to 1V209. Representative TEM images of (B) SaPAC(ii)‐OVA and (C) SaPAC(iii)‐OVA nanoparticles. (D) Prime‐boost vaccination schedule. (E, F) IFN‐gamma ELISPOT images and quantification. (G, H) CD69 expression on CD8^+^ and CD4^+^ T cells after ex vivo OVA restimulation. (I) Cytokine profiling of culture supernatants after 24 h restimulation. Data are the mean ± SEM; significance was assessed by unpaired two‐tailed Student's t‐test (^*^ *p* < 0.05, ^**^ *p* < 0.01, ^***^ *p* < 0.001).

Physicochemical characterization by TEM confirmed that nanoparticle self‐assembly was preserved after 1V209 conjugation (Figure 3B,C). Representative NTA size distributions for SaPN‐OVA and SaPAC‐OVA are shown in Figure S4C,D, and the corresponding cross‐construct hydrodynamic diameter and zeta‐potential summary is shown in Figure S4E and Table S2. NTA/ZetaView measurements showed hydrated diameters in the 100–200 nm range across constructs, including SaPAC‐OVA at 169 ± 18 nm, and cationic zeta potentials. TEM images appeared smaller than NTA/ZetaView sizes because TEM captures dry‐state primary structures, whereas NTA/ZetaView reports hydrodynamic behavior in buffer.

We next evaluated these variants using a prime‐boost regimen in female C57BL/6J mice (Figure 3D–F). ELISPOT analysis of splenocytes restimulated with SIINFEKL showed that SaPAC‐OVA induced the highest frequency of IFN‐gamma‐secreting T cells (Figure 3E,F). Covalent tethering of 1V209 was essential, because the molar‐matched unconjugated nanoparticle control (SaPN‐OVA + 1V209) produced only marginal improvement over soluble peptide plus adjuvant.

Flow cytometric analysis after 24 h ex vivo restimulation showed that SaPAC‐OVA increased CD69 expression on both CD8^+^ effector T cells and CD4^+^ helper T cells (Figure 3G,H). Cytokine profiling indicated a robust but balanced inflammatory signature, including elevated TNF‐alpha and IL‐1alpha (Figure 3I and Figure S8). Poly(I:C) was used as a widely adopted preclinical/clinical benchmark adjuvant rather than a mechanistically equivalent control, because Poly(I:C) primarily activates dsRNA‐sensing pathways such as TLR3 and MDA5, whereas SaPAC engages TLR7‐MyD88 signaling through tethered 1V209. Compared with Poly(I:C)‐adjuvanted groups, SaPAC‐OVA maintained IL‐6 concentrations near baseline.

### 2.6 Transcriptomic Profiling Reveals SaPAC Immunogenicity is Driven by TLR–MyD88‐Dependent Innate Activation

To probe the mechanistic basis of SaPAC‐OVA immunogenicity, we performed bulk RNA‐seq profiling of draining LN cells after immunization with SaPAC‐OVA or component controls (OVA + 1V209 and SaPN‐OVA + 1V209). Each treatment induced a distinct transcriptional program relative to Mock controls (Figure 4A). SaPAC‐OVA uniquely upregulated 633 genes, consistent with a conjugation‐dependent effect beyond simple co‐administration. GO enrichment highlighted immune signaling regulation, cytokine production, leukocyte activation, phagocytosis‐related genes, and TLR pathway genes (Figure 4B–D). In MB49 tumor‐draining LNs, SaPAC‐MB49 induced a similar transcriptional profile (Figure S9A–D).

**FIGURE 4:**
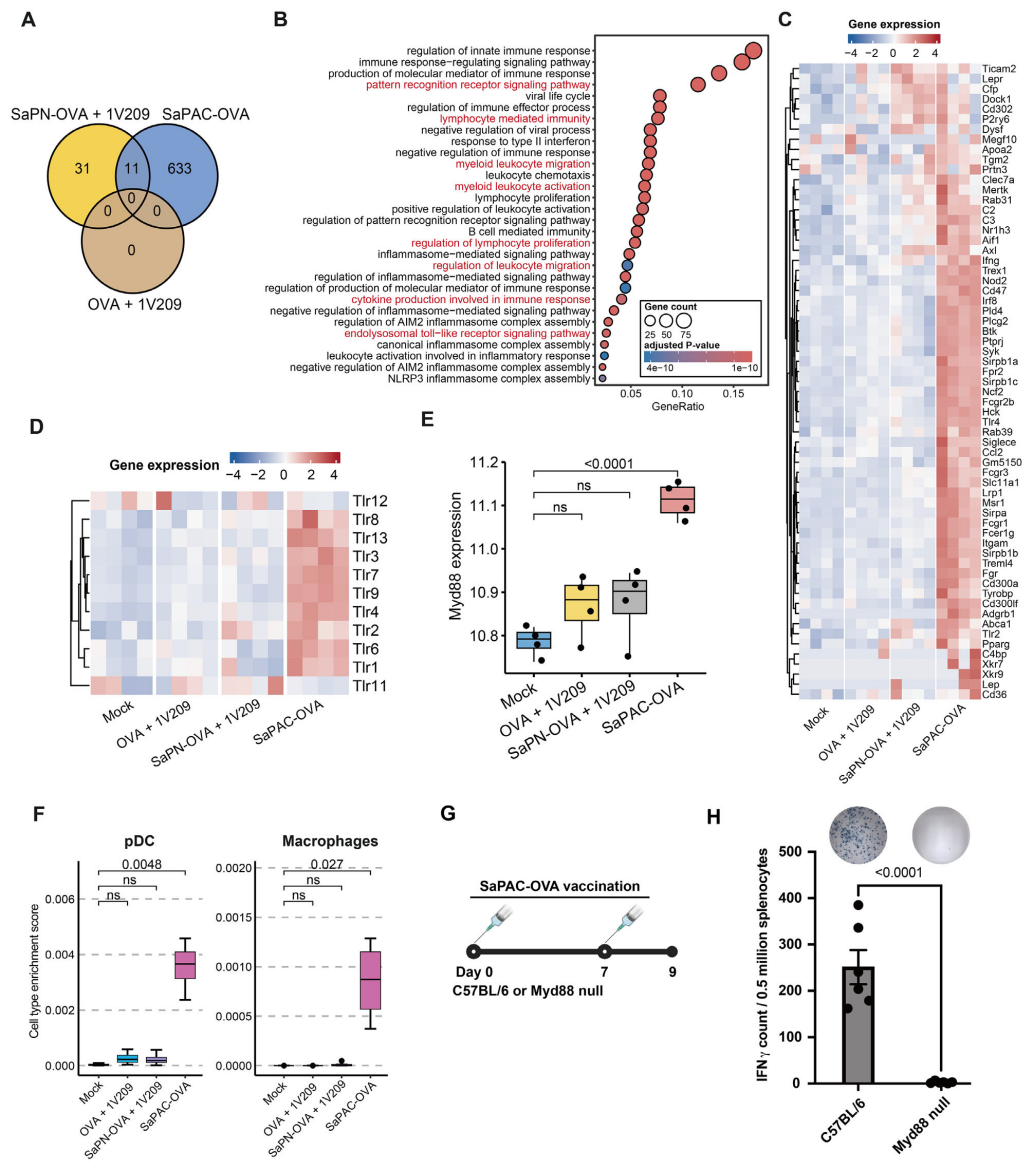
Transcriptomic analysis of innate immune activation by SaPAC nanoparticles and MyD88‐dependent in vivo immunogenicity. (A) Venn diagram of significantly upregulated genes. (B) GO enrichment analysis. (C) Heatmap of phagocytosis‐related genes. (D) Heatmap of TLR family gene expression. (E) MyD88 expression in LN cells. (F) ssGSEA scores for plasmacytoid DCs and macrophages. (G) Vaccination schedule in C57BL/6J and Myd88‐null mice. (H) IFN‐gamma ELISPOT images and quantification. Data are the mean ± SEM; significance was assessed by unpaired two‐tailed Student's t‐test (^*^ *p* < 0.05, ^**^ *p* < 0.01, ^***^ *p* < 0.001).

This conjugate‐specific transcriptional signature supports targeted engagement of TLR7 by covalently linked 1V209. MyD88 is a central adaptor for most TLR pathways and connects activated receptors to IRAK/TRAF6‐mediated inflammatory signaling. MyD88 expression was increased in LNs from SaPAC‐OVA‐treated mice (Figure 4E), prompting functional testing in MyD88‐null mice. Parallel vaccination of C57BL/6J and Myd88‐null mice showed that the enhanced IFN‐gamma ELISPOT response induced by SaPAC‐OVA was lost in the absence of MyD88 (Figure 4G,H), establishing SaPAC immunogenicity as TLR‐MyD88 dependent. ssGSEA further showed enrichment of plasmacytoid DC and macrophage signatures after SaPAC‐OVA administration (Figure 4F) [31].

### 2.7 SaPAC Nanoparticles Drive Intratumoral CD8^+^ T‐Cell Infiltration and Antitumor Efficacy Across Diverse Cancer Models

Poly(I:C) is widely used as a benchmark adjuvant for neoantigen screening and vaccine studies and has been evaluated in early‐phase clinical trials [32, 33]. We therefore used Poly(I:C) as a positive benchmark rather than a mechanistically equivalent comparator, because Poly(I:C) activates dsRNA‐sensing pathways such as TLR3/MDA5, whereas SaPAC uses covalently tethered 1V209 to engage TLR7‐MyD88 signaling. In the subcutaneous B16‐OVA model, SaPAC‐OVA markedly suppressed tumor growth and increased activated CD8^+^ T‐cell and NK‐cell infiltration (Figure 5A–F and Figure S10).

**FIGURE 5:**
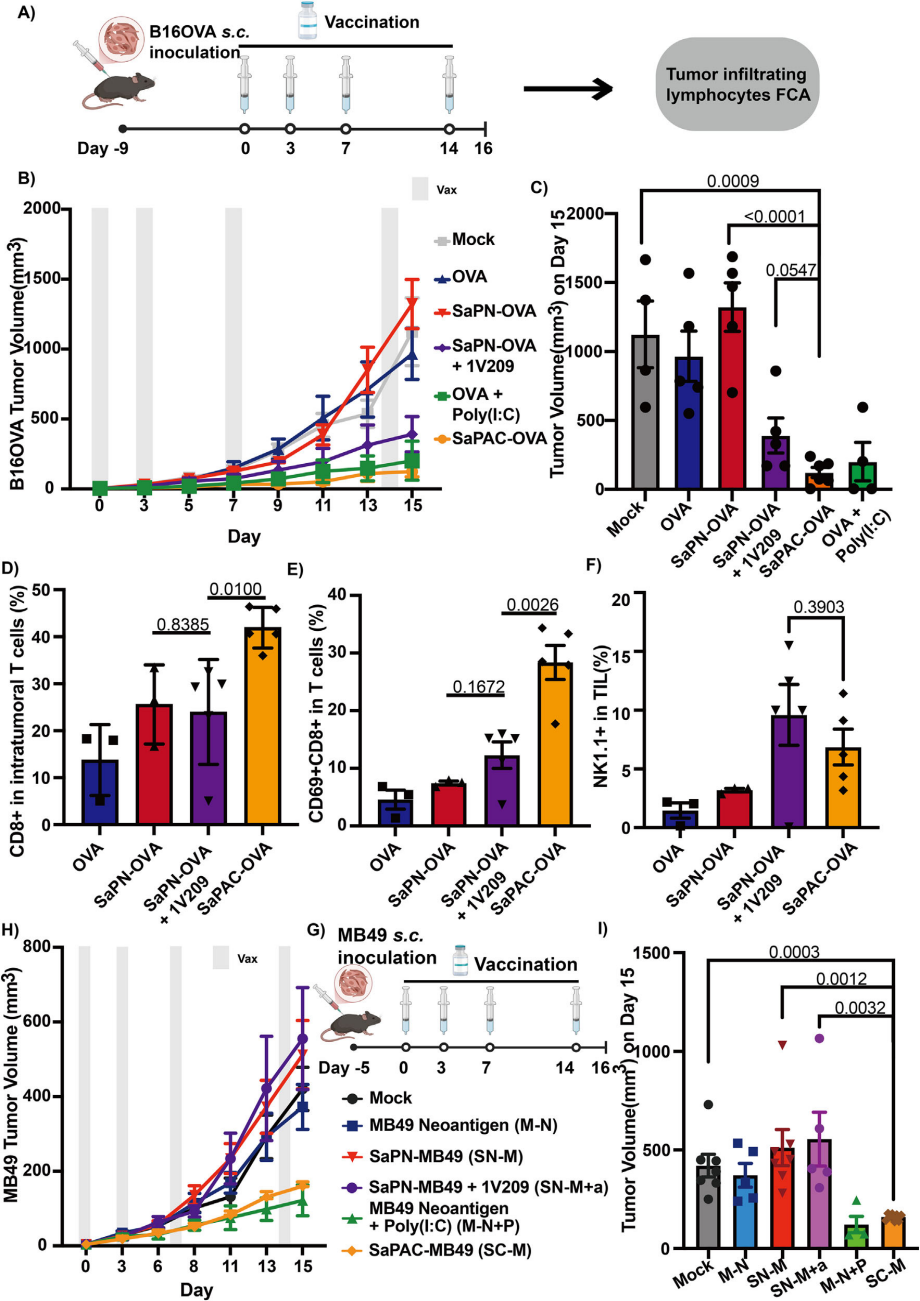
Antitumor efficacy of SaPAC nanoparticles carrying OVA or MB49 neoantigens. (A) B16‐OVA melanoma treatment timeline. (B) Longitudinal B16‐OVA tumor volumes (*n* = 4–6 mice/group). (C) Tumor volumes on day 15. (D–F) Endpoint flow cytometric analysis of B16‐OVA tumor‐infiltrating lymphocytes: CD8^+^ T‐cell frequency, CD69 expression on CD8^+^ T cells, and CD3^−^NK1.1^+^ NK‐cell frequency. (G) MB49 bladder carcinoma treatment timeline. (H) Longitudinal MB49 tumor volumes (*n* = 4–8 mice/group). (I) Tumor volumes on day 15. Data are the mean ± SEM; significance was assessed by unpaired two‐tailed Student's t‐test (^*^ *p* < 0.05, ^**^ *p* < 0.01, ^***^ *p* < 0.001).

To assess versatility across tumor types, we generated SaPAC‐MB49 using a lab‐screened MB49 neoantigen (sequence and screening summary in Table S1, Figures S11 and S12). Mice received 2 × 10^−8^ mol antigen per group as MB49 neoantigen peptide, SaPN‐MB49, SaPN‐MB49 + 1V209, MB49 neoantigen + Poly(I:C), or SaPAC‐MB49 on days 0, 3, 7, and 14. SaPAC‐MB49 contains three covalently linked 1V209 moieties per construct; the SaPN‐MB49 + 1V209 group therefore received 6 × 10^−8^ mol unconjugated 1V209, corresponding to three molar equivalents per antigen molecule. SaPAC‐MB49 reduced endpoint tumor volumes to <200 mm^3^ on day 15, comparable to the Poly(I:C)‐adjuvanted benchmark, whereas unconjugated controls showed limited therapeutic benefit (Figure 5H,I and Figure S12).

### 2.8 Multivalent SaPAC Synergizes With PD‐1 Blockade to Overcome Immunosuppression in Cold TNBC Tumors

In the MB49 model, SaPAC‐MB49 treatment increased PD‐1 expression on T cells in tumor‐draining LNs at later treatment stages (Figure S9E). This finding suggests that adaptive immune resistance may emerge during prolonged vaccine‐driven T‐cell activation [34], providing a rationale for combining SaPAC vaccination with PD‐1 blockade.

Given that α‐PD‐1 immune checkpoint blockade functions as a cornerstone immunotherapy by disrupting PD‐1/PD‐L1 interactions and thereby restoring T‐cell effector function, we sought to evaluate the potential synergistic effect of combining SaPAC with α‐PD‐1 therapy. To this end, we extended the application of SaPAC to an immunologically cold tumor model—triple‐negative breast cancer (TNBC)—which is characterized by a highly immunosuppressive tumor microenvironment. We hypothesized that a multivalent peptide vaccine formulation would enhance the induction of cellular immunity, and that its combination with α‐PD‐1 blockade would yield improved therapeutic efficacy. Accordingly, we employed the orthotopic 4T1 TNBC model for subsequent investigation.

For the 4T1 model, WES/RNA‐seq identified somatic mutations and NetMHCpan [35] prioritized candidate neoantigens (Figure S13A). Candidate peptides were screened with Poly(I:C)‐adjuvanted vaccination and ELISPOT validation (Figure S13B). Three hits, 4T1#2, 4T1#3, and 4T1#13, were selected for SaPAC conjugation to generate triSaPAC‐4T1 (Figure S13C and Table S1). Wild‐type counterpart peptides did not show cross‐reactive immunogenicity (Figure S13D). As monotherapy, triSaPAC‐4T1 showed suboptimal tumor suppression compared with the Poly(I:C)‐adjuvanted benchmark (Figure S14), supporting evaluation with anti‐PD‐1.

We then evaluated triSaPAC‐4T1 plus anti‐PD‐1 in orthotopic 4T1 TNBC. Female BALB/c mice were inoculated in the mammary fat pad with 1 × 10^5^ 4T1 cells on day ‐5. When tumors reached approximately 25 mm^3^ on day 0, mice were randomized and vaccinated on days 0, 3, and 7 with mock, triple‐valent 4T1 neoantigens(4T1#2, 4T1#3, and 4T1#13 mixture) + Poly(I:C), or triSaPAC‐4T1. Anti‐PD‐1 antibody was administered intraperitoneally every 2–3 days, and tumors were monitored until day 16 (Figure 6A).

**FIGURE 6:**
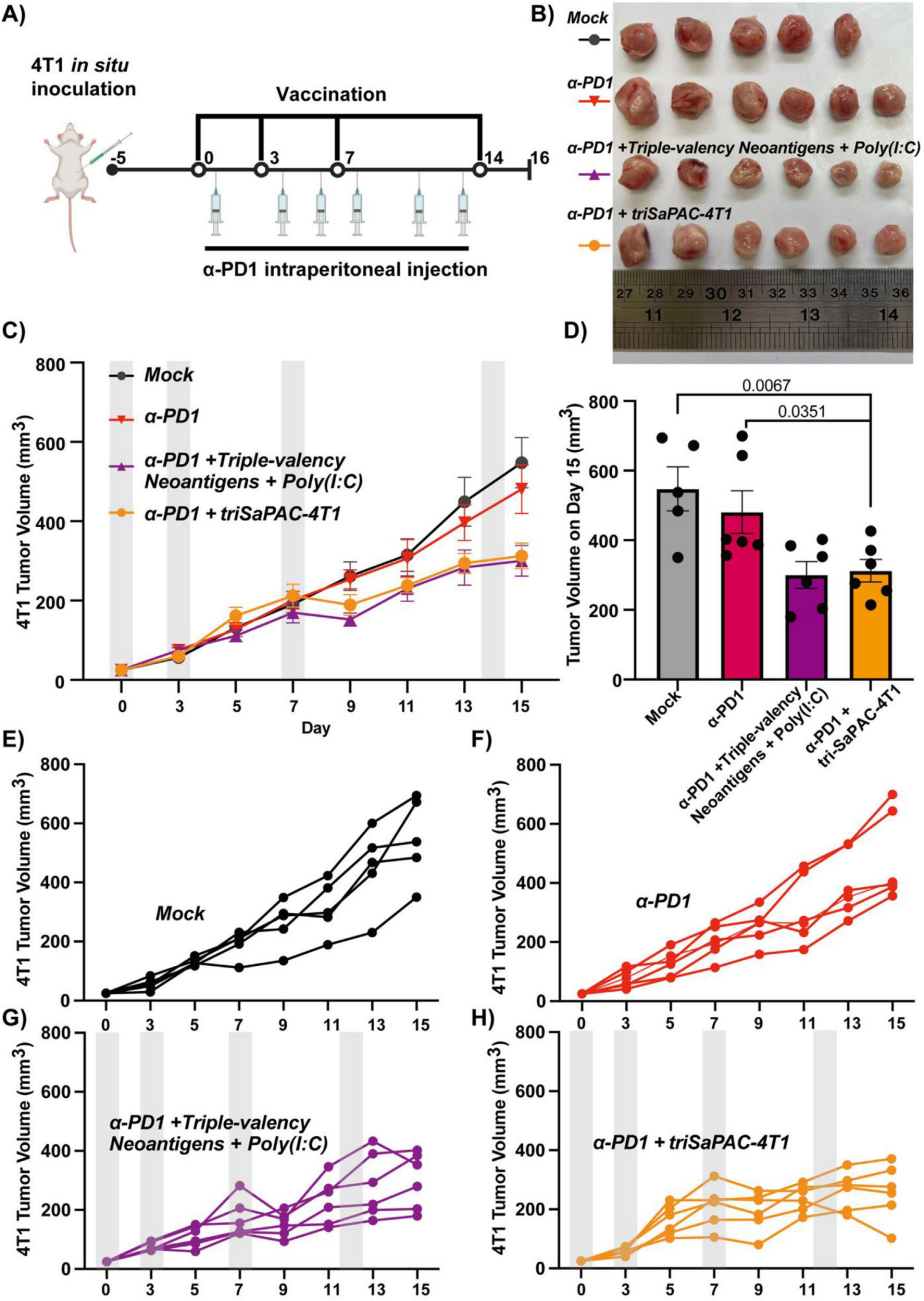
Synergistic antitumor efficacy of trivalent SaPAC‐4T1 vaccination combined with anti‐PD‐1 checkpoint blockade in orthotopic 4T1 TNBC. (A) Treatment timeline. (B) Representative endpoint tumors (day 16; scale bar, 10 mm). (C) Longitudinal tumor volumes (*n* = 6–8 mice/group). (D) Terminal tumor volumes on day 15. (E,F) Individual tumor growth trajectories. Data are the mean ± SEM; significance was assessed by unpaired two‐tailed Student's t‐test (^*^ *p* <0.05, ^**^ *p* <0.01, ^***^ *p* <0.001 vs. mock + anti‐PD‐1).

The triple 4T1 neoantigens + Poly(I:C) + anti‐PD‐1 group served as a positive benchmark for neoantigen‐specific efficacy. Anti‐PD‐1 monotherapy showed minimal activity, whereas triSaPAC‐4T1 + anti‐PD‐1 produced robust and sustained suppression from day 9 onward, with endpoint tumor volumes comparable to the Poly(I:C)‐adjuvanted benchmark (Figure 6C,D,G,H).

Together, the B16‐OVA, MB49, and 4T1 studies show that SaPAC can be adapted from model antigens to tumor neoantigens and can function either as monotherapy in responsive models or as a combination partner for checkpoint blockade in cold tumors.

## 3 Discussion

The development of effective cancer vaccines requires coordinated antigen delivery, innate immune activation, and durable T‐cell priming. Here, we introduce SaPAC, a modular self‐assembling peptide‐adjuvant conjugate platform that covalently links neoantigen epitopes to the TLR7 agonist 1V209 within cationic nanoparticles. SaPAC improved accumulation and retention in draining LNs, enhanced DC uptake and MHC‐I cross‐presentation, activated MyD88‐dependent innate signaling, and generated antitumor activity across B16‐OVA melanoma, MB49 bladder carcinoma, and orthotopic 4T1 TNBC models.

As summarized in Figure 7, SaPAC acts by co‐localizing antigen and adjuvant in lymphoid tissues. After subcutaneous injection, nanoparticles drain to LNs, are internalized by APCs, and promote DC maturation. Processed neoantigen peptides are then presented to CD8^+^ and CD4^+^ T cells, generating systemic antitumor responses capable of infiltrating tumors and mediating cytotoxic activity.

**FIGURE 7:**
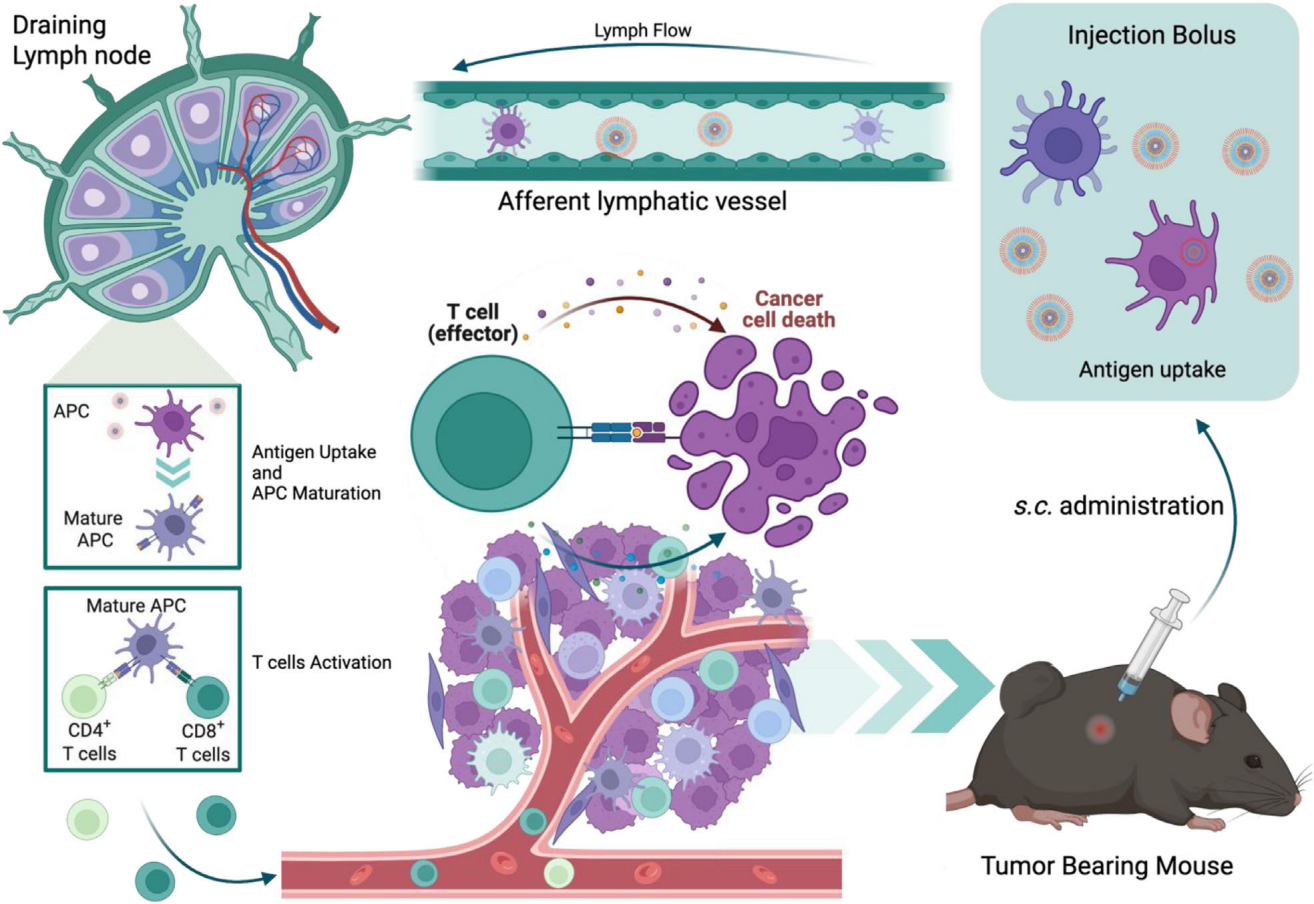
Mechanistic overview of SaPAC‐mediated antitumor immunity. After subcutaneous administration, self‐assembled SaPAC nanoparticles drain through lymphatic capillaries and accumulate in proximal draining lymph nodes. Dendritic cells internalize the nanoparticles, process the linked neoantigen for MHC‐restricted presentation, and receive synchronized TLR7 stimulation from tethered 1V209. Mature DCs then prime antigen‐specific CD8^+^ and CD4^+^ T cells, supporting clonal expansion, systemic circulation of effector cells, tumor infiltration, and tumor cell elimination.

These findings extend prior peptide–TLR ligand conjugate and self‐assembling peptide‐adjuvant vaccine work by emphasizing chemically defined, site‐selective antigen–adjuvant stoichiometry and modular neoantigen replacement [18, 20, 24]. Unlike simple mixtures of peptide and soluble TLR agonist, SaPAC preserves antigen–adjuvant co‐delivery during lymphatic trafficking and cellular uptake. Compared with liposomal or viral delivery systems, short peptide conjugates may offer simpler chemical quality control, scalable synthesis, and rapid adaptation to selected epitopes.

The observed enrichment of plasmacytoid DCs and macrophages in LNs highlights the platform's ability to engage multiple innate immune cell types while supporting adaptive effector responses. In B16‐OVA tumors, SaPAC‐OVA increased activated CD8^+^ T‐cell and NK‐cell infiltration. In MB49 tumors, SaPAC‐MB49 suppressed tumor growth comparably to the Poly(I:C)‐adjuvanted benchmark. In orthotopic 4T1 TNBC, triSaPAC‐4T1 required anti‐PD‐1 combination therapy to achieve durable tumor suppression, illustrating how SaPAC can be paired with checkpoint blockade in immunologically cold settings.

## 4 Study Limitations and Future Directions

The study has several limitations. First, B16‐OVA is an engineered, highly immunogenic model and may overestimate responses achievable with endogenous human neoantigens. Second, although MB49 and 4T1 experiments extend the platform to tumor neoantigens, broader validation in additional syngeneic, humanized, and patient‐derived models will be needed. Third, lymph node retention was inferred primarily from fluorescence imaging and short‐term pharmacokinetic modeling; longer longitudinal biodistribution, degradation, and dose‐ranging studies remain important. Fourth, SaPAC uses TLR7‐MyD88 signaling, whereas patient tumors may require alternative or multi‐agonist innate programs. Finally, clinical translation will require GMP synthesis, batch‐to‐batch physicochemical comparability, toxicology, and formal safety testing.

## 5 Comparison With mRNA‐LNP Neoantigen Vaccines

SaPAC complements, rather than replaces, emerging personalized mRNA–LNP neoantigen vaccines. Landmark clinical studies have shown that individualized RNA/mRNA neoantigen vaccines can mobilize poly‐specific T‐cell responses in melanoma and can induce neoantigen‐specific CD8^+^ T cells associated with delayed recurrence in pancreatic cancer [36, 37]. More recently, adjuvant mRNA‐4157 (V940) plus pembrolizumab improved recurrence‐free survival vs. pembrolizumab alone in resected high‐risk melanoma in the phase 2b KEYNOTE‐942 study [38]. mRNA platforms can encode multiple long antigens and may generate broad CD4^+^ and CD8^+^ responses after intracellular antigen expression. SaPAC instead directly loads selected peptide epitopes, fixes antigen/adjuvant stoichiometry chemically, and may simplify quality control for short personalized constructs. These features could support rapid manufacturing for patients whose actionable epitopes are already defined, while mRNA–LNP approaches may remain advantageous when broader antigen encoding is desired.

Beyond oncology, the modular and chemically defined nature of SaPAC could be adapted to infectious disease vaccines that require durable T‐cell immunity. The same scaffold logic may also be repurposed for tolerogenic applications by replacing the TLR agonist with immunoregulatory cargo designed to promote antigen‐specific tolerance in autoimmunity or allergy. More broadly, self‐assembling peptide conjugation may improve the pharmacokinetics and tissue exposure of peptide therapeutics by protecting cargo from proteolysis, increasing local retention, and enabling depot‐like release.

## 6 Conclusion

In summary, SaPAC is a chemically defined cancer vaccine scaffold that integrates self‐assembly, lymph node‐targeted delivery, TLR7 adjuvanticity, and neoantigen presentation to elicit T‐cell immunity. By promoting DC maturation, LN retention, and tumor‐infiltrating effector lymphocytes, SaPAC suppressed tumor growth across melanoma, bladder carcinoma, and breast cancer models, particularly when combined with immune checkpoint blockade. This modular platform may support precision oncology strategies that require rapid adaptation to patient‐specific neoantigens.

## 7 Materials and Methods

### 7.1 Cell lines

The DC2.4 cell line, mouse bladder cancer cell line MB49, mouse melanoma cell line B16F10, and the mouse breast cancer cell line 4T1 were purchased from ATCC. The mouse melanoma B16F10‐OVA cell line was previously developed by our Lab. The MB49 cell line was cultured in Dulbecco's Modified Eagle Medium (DMEM) with 10% Fetal Bovine Serum (FBS) and 1% Penicillin and Streptomycin (PS). The DC2.4 cell line, 4T1 cell line, and B16F10‐OVA were cultured in Roswell Park Memorial Institute (RPMI) 1640 medium with 10% Fetal Bovine Serum (FBS) and 1% Penicillin and Streptomycin (PS). All cell lines are cultured at 37°C in 5% *CO*_2_ incubator.

### 7.2 Mice

C57BL/6J and BALB/c were purchased from CCMR. Both mouse strains were used between 6 and 8 weeks of age unless otherwise noted. All animal work was conducted under the CULATR #23‐299, #23‐274, and #5921‐20.

### 7.3 Peptides and Adjuvants

All peptides, peptide‐adjuvant conjugates, and fluorescently labeled peptides were synthesized by GL Biochem (Shanghai) Ltd. and are listed in Table 1. In these sequences, the antigen epitope is positioned between SLVR cathepsin‐sensitive linkers; N‐terminal lysines contribute cationic charge and self‐assembly; K^*^ indicates lysine side chains conjugated to 1V209; and SaPN constructs lack covalently attached 1V209. Unconjugated 1V209 used in physical‐mixture controls was obtained from MedChemExpress (HY‐115400). Poly(I:C) (HMW) was purchased from InvivoGen and used as a benchmark adjuvant control. Fluorescent peptides were dissolved in PBS containing 1% DMSO.

**TABLE 1:** Peptides, peptide‐adjuvant conjugates, and fluorescently labeled peptides used in this study. The antigen epitope, linker/self‐assembly residues, and 1V209‐conjugated lysines (K^*^) are indicated in the sequence column.

Index	Peptide sequence (Left: N‐terminus)	Post‐modifications	Purity
Ovalbumin 257–264 (OVA)	SIINFEKL	Nil	95%
FITC‐OVA epitope (FITC‐OVA)	FITC‐SIINFEKL	FITC‐: FITC‐labelled N‐terminus	95%
Cy3‐OVA epitope (Cy3‐OVA)	Cy3‐ SIINFEKL	Cy3‐: Cy3 labelled N terminus	95%
SaPAC(ii)‐OVA	KKKKK SLVR SIINFEKL SLVR K^*^K^*^K^*^	K^*^: 1V209 conjugation‐lysine	95%
SaPAC‐OVA	KKKKK SLVR SIINFEKL SLVR K^*^AK^*^AK^*^	K^*^: 1V209 conjugation‐lysine	95%
Cy3‐SaPAC‐OVA	Cy3‐ KKKKK SLVR SIINFEKL SLVR K^*^AK^*^AK^*^	(1) K^*^: 1V209 conjugation‐lysine (2) Cy3‐: Cy3 labelled N terminus	95%
SaPAC(iii)‐OVA	KKKKK SLVR SIINFEKL SLVR K^*^WK^*^WK^*^	K^*^: 1V209 conjugation‐lysine	95%
SaPN‐OVA	KKKKK SLVR SIINFEKL SLVR	Nil	95%
FITC‐SaPN‐OVA	FITC‐ KKKKK SLVR SIINFEKL SLVR	FITC‐: FITC‐labelled N‐terminus	95%
Cy3‐SaPN‐OVA	Cy3‐ KKKKK SLVR SIINFEKL SLVR	Cy3‐: Cy3‐labelled N‐terminus	95%
Synthetic Peptide MB49‐1 (MB49 Neoantigen)	SLRVVAYSLRTALAFCSPRPCVPSAAA	Nil	95%
Synthetic Peptide MB49‐2 (SaPN‐MB49)	KKKKK SLVR SLRVVAYSLRTALAFCSPRPCVPSAAA SLVR	Nil	95%
Synthetic Peptide MB49‐3 (SaPAC‐MB49)	KKKKK SLVR SLRVVAYSLRTALAFCSPRPCVPSAAA SLVR K^*^AK^*^AK^*^	K^*^: 1V209 conjugation‐lysine	95%
Synthetic Peptide 4T1‐1 (4T1#1)	MATEIGSPPRFFYMPRFQHQAPRQL	Nil	95%
Synthetic Peptide 4T1‐2 (4T1#2)	AEESEIRYSTWKRAVMKSIGWVTTQ	Nil	95%
Synthetic Peptide 4T1‐3 (4T1#3)	FVQWKVVERLDKQTEIYHYVLNSMV	Nil	95%
Synthetic Peptide 4T1‐4 (4T1#4)	REENVKADVFHAYLSLLTQTCPVQS	Nil	95%
Synthetic Peptide 4T1‐5 (4T1#5)	SPSLSGQQEIFTHIMDHYSYCTPSH	Nil	95%
Synthetic Peptide 4T1‐6 (4T1#6)	GSSTPMFSMSSPISRRFNNLFGK	Nil	95%
Synthetic Peptide 4T1‐7 (4T1#7)	RKSSGLIVSKAPHNFQFVQKPDESG	Nil	95%
Synthetic Peptide 4T1‐8 (4T1#8)	TEENKISKTELDWFFQDLDREIKKW	Nil	95%
Synthetic Peptide 4T1‐9 (4T1#9)	LETPRGKIQAKKWSLVPFSIPVFDI	Nil	95%
Synthetic Peptide 4T1‐10 (4T1#10)	RGREQSLQCHYPTEHCIEVVTLQST	Nil	95%
Synthetic Peptide 4T1‐11 (4T1#11)	FSCLLAHALNLIKLVRGRKPLSWLV	Nil	95%
Synthetic Peptide 4T1‐12 (4T1#12)	MELFRVCLVVVTGIINHPLLFPREN	Nil	95%
Synthetic Peptide 4T1‐13 (4T1#)	ADAYAAHIRGAKHQKVVTLHTKLGK	Nil	95%
Synthetic Peptide 4T1‐14 (4T1#14)	VESKYTVENGYDGNAKVVYGDTDSV	Nil	95%
Synthetic Peptide 4T1‐2WT (WT#2)	AEESEIRYSTWKKAVMKSIGWVTTQ	Nil	95%
Synthetic Peptide 4T1‐3WT (WT#3)	FVQWKVVERLDKQTEIYQYVLNSMV	Nil	95%
Synthetic Peptide 4T1‐13WT (WT#13)	ADAYAAHIRGAKHQKVVKLHTKLGK	Nil	95%
Synthetic Peptide 4T1‐15 (SaPAC‐4T1#2)	KKKKKSLVRAEESEIRYSTWKRAVMKSIGWVTTQSLVRK^*^AK^*^AK^*^	K^*^: 1V209 conjugation‐lysine	95%
Synthetic Peptide 4T1‐16 (SaPAC‐4T1#3)	KKKKKSLVRFVQWKVVERLDKQTEIYHYVLNSMVSLVRK^*^AK^*^AK^*^	K^*^: 1V209 conjugation‐lysine	95%
Synthetic Peptide 4T1‐17 (SaPAC‐4T1#13)	KKKKKSLVRADAYAAHIRGAKHQKVVTLHTKLGKSLVRK^*^AK^*^AK^*^	K^*^: 1V209 conjugation‐lysine	95%

### 7.4 Transmission Electron Microscopy and ZetaView/NTA Measurement of Nanoparticles

SaPAC(ii)‐OVA, SaPAC(iii)‐OVA, SaPAC‐OVA, SaPN‐OVA, SaPAC‐MB49, SaPAC‐4T1#2, SaPAC‐4T1#3, and SaPAC‐4T1#13 were prepared at the indicated concentrations and imaged by Philips CM100 TEM with an Olympus SIS Tengra CCD camera. Hydrodynamic diameter and zeta potential were measured using a ZetaView PMX120 Nanoparticle Tracking Analyzer. SaPAC(ii)‐OVA, SaPAC(iii)‐OVA, SaPAC‐OVA, and SaPN‐OVA were analyzed at 5 µg/mL; SaPAC‐4T1#2 at 10 µg/mL; SaPAC‐MB49, SaPAC‐4T1#3, and SaPAC‐4T1#13 at 30 µg/mL. Consolidated size, zeta‐potential, concentration, buffer, and batch information are summarized in Table S2.

### 7.5 Tumor Infiltrating Lymphocytes Preparation for Flow Cytometry Analysis

Tumors were dissected from mice and digested with DNAse I (0.1 mg/mL, 11284932001, Roche) and Collagenase/Hyaluronidase (07912, Stemcell Technologies) for 45 min to collect all cells. Then, cells were filtered through a 70 µm cell strainer and collected by centrifuge under 550 g for 5 min. The TILs, along with tumor cells, were washed with PBS twice, and cells were blocked with Trypsin (Bolegend) on ice for 20 min. All cells were collected by centrifuge under 550 g for 5 min, and then were stained with an antibody mixture (surface biomarker) on ice for 40 min. The stained cells were washed with PBS twice, and then the cells were resuspended in a flow buffer (PBS with 0.5% FBS). For the intracellular staining of cells, the cells were fixed and permeabilized according to the protocol from the Fixation/Permeabilization Solution Kit (BD Biosciences, Cat. # 554714). After fixation and permeabilization, the cells were stained with the intracellular antibody mixture on ice for 40 min. Surface and intracellular staining reagents are listed in Table 2. After being washed with PBS, the cells were resuspended in the flow buffer and then assessed by flow cytometry (ACEA Novocyte Quanteon) from the Centre for PanorOmic Sciences, Li Ka Shing Faculty of Medicine, The University of Hong Kong.

### 7.6 Bone Marrow Derived Dendritic Cells Preparation

Bone marrow‐derived cells were taken out from tibial fractures of C57BL/6J mice. All bone marrow‐derived cells were cultured in RPMI 1640 (with 10% FBS and 1% P/S) containing granulocyte‐macrophage colony‐stimulating factor (final concentration: 40 ng/mL, Human GM‐CSF Recombinant Protein, Cat # 300‐03‐100UG) and mouse interleukin‐4 (final concentration: 20 ng/mL, Sino Biological, Cat # 51084‐MNAE‐20) [29]. The media was changed every 2 days. After 8 days of incubation and differentiation, all low‐attachment cells and high‐attachment cells were collected. 500 000 cells of the cell mixture (both low‐attachment cells and high‐attachment cells) were added into each well of a 24‐well cell culture plate along with 1 nmol SaPAC‐OVA, 1 nmol SaPN‐OVA, 1 nmol SaPN‐OVA, along with 3 nmol 1V209 and 1 nmol OVA epitope. After 24 h of co‐culture, all low‐attachment cells and high‐attachment cells were collected. All cells were then washed with PBS twice. Cells were stained with antibodies and assessed by flow cytometry (ACEA Novocyte Quanteon) from the Centre for PanorOmic Sciences, Li Ka Shing Faculty of Medicine, The University of Hong Kong.

### 7.7 Tumor Inoculation and Therapeutic Treatment

#### 7.7.1 B16‐OVA Tumor Inoculation

B16‐OVA cells were injected subcutaneously into female C57BL/6J mice on day ‐9. Mice were randomized on day 0 and vaccinated subcutaneously with 2.5 × 10^−8^ mol antigen per group as SaPAC‐OVA, SaPN‐OVA, OVA, or the corresponding controls. SaPAC‐OVA contains three 1V209‐conjugated lysines; therefore, SaPN‐OVA + 1V209 received 7.5 × 10^−8^ mol unconjugated 1V209, corresponding to three molar equivalents per antigen molecule. The Poly(I:C) group received 2.5 × 10^−8^ mol OVA plus 250 µg Poly(I:C) as a mechanistically distinct benchmark adjuvant control. Tumor volumes were measured every 2–3 days, and mice were sacrificed on day 16 for flow cytometric analysis.

#### 7.7.2 MB49 Tumor Inoculation

MB49 cells were injected subcutaneously into male C57BL/6J mice on day ‐5. Mice were randomized on day 0 and vaccinated subcutaneously on days 0, 3, 7, and 14 with 2 × 10^−8^ mol antigen per group as SaPAC‐MB49, SaPN‐MB49, MB49 neoantigen, or the corresponding controls. SaPAC‐MB49 contains three 1V209‐conjugated lysines; therefore, SaPN‐MB49 + 1V209 received 6 × 10^−8^ mol unconjugated 1V209, corresponding to three molar equivalents per antigen molecule. The Poly(I:C) group received 2 × 10^−8^ mol MB49 neoantigen plus 250 µg Poly(I:C) as a mechanistically distinct benchmark adjuvant control. Tumor volumes were measured every 2–3 days, and mice were sacrificed on day 16.

#### 7.7.3 4T1 Tumor Inoculation

4T1 cells were injected into the mammary fat pad of female BALB/c mice on day ‐5. Mice were randomized on day 0 and vaccinated subcutaneously on days 0, 3, 7, and 14 with triSaPAC‐4T1 (1.65 × 10^−8^ mol SaPAC‐4T1#2, 1.79 × 10^−8^ mol SaPAC‐4T1#3, and 1.72 × 10^−8^ mol SaPAC‐4T1#13), an unconjugated 4T1 neoantigen mixture at matched peptide doses (1.65 × 10^−8^ mol 4T1#2, 1.79 × 10^−8^ mol 4T1#3, and 1.72 × 10^−8^ mol 4T1#13) plus unconjugated 1V209 (1.55 × 10^−7^ mol), or the matched 4T1 neoantigen mixture (1.65 × 10^−8^ mol 4T1#2, 1.79 × 10^−8^ mol 4T1#3, and 1.72 × 10^−8^ mol 4T1#13) plus 250 µg Poly(I:C). The total triSaPAC‐4T1 dose was 5.16 × 10^−8^ mol. Because each SaPAC construct carries three 1V209 moieties, the free‐1V209 control received 1.55 × 10^−7 ^mol, corresponding to three molar equivalents relative to the total peptide‐conjugate dose. Tumor volumes were measured every 2–3 days, and mice were sacrificed on day 16.

#### 7.7.4 4T1 Tumor Combinational Immunotherapy

For 4T1 combination immunotherapy, tumor‐bearing female BALB/c mice were randomized on day 0 and treated with triple‐valent 4T1 neoantigen mixtures (1.65 × 10^−8^ mol 4T1#2, 1.79 × 10^−8^ mol 4T1#3, and 1.72 × 10^−8^ mol 4T1#13), triSaPAC‐4T1 (1.65 × 10^−8^ mol SaPAC‐4T1#2, 1.79 × 10^−8^ mol SaPAC‐4T1#3, and 1.72 × 10^−8^ mol SaPAC‐4T1#13), anti‐PD‐1, or triSaPAC‐4T1 (1.65 × 10^−8^ mol SaPAC‐4T1#2, 1.79 × 10^−8^ mol SaPAC‐4T1#3, and 1.72 × 10^−8^ mol SaPAC‐4T1#13) plus anti‐PD‐1. Anti‐PD‐1 antibody (InVivoMAb anti‐mouse PD‐1/CD279, BE0146) was administered intraperitoneally at 200 µg per dose on days 1, 4, 6, 9, 11, and 13. Tumor volumes were measured every 2–3 days, and mice were sacrificed on day 16.

**TABLE 2:** Reagents for flow cytometry analysis. All antibodies for both intracellular staining and surface immunostaining, viability dyes, and compensation beads were included in the table.

Antibody/Dye/Compensation bead	Source	Cat. #
TruStain FcX PLUS anti‐mouse CD16/32 Antibody	Biolegend	156604
Fixable Viability Dye eFluor 780	Biolegend	65‐0865‐14
PE anti‐mouse H‐2K^b^‐bound to SIINFEKL Antibody	Biolegend	141603
Anti‐CD45 Rat Monoclonal Antibody (Alexa Fluor 594) [clone: 30‐F11], Size = 100 µg	BioLegend	103144
FITC anti‐mouse CD3 [17A2] 500 µg	Biolegend	100204
PerCP/Cy5.5 anti‐mouse CD8a [53‐6.7] 25 µg	Biolegend	100733
Brilliant Violet 711 anti‐mouse CD4 [RM4‐5] 50 µg	Biolegend	100557
PE/Cy7 anti‐mouse/human CD44 [IM7] 100 µg	Biolegend	103030
Brilliant Violet 421 anti‐mouse CD69 [H1.2F3] 50 ug	Biolegend	104545
Anti‐CD25 Rat Monoclonal Antibody (Brilliant Violet 605) [clone: PC61], Size = 125 µL	Biolegend	102035
Alexa Fluor 700 anti‐mouse IFN‐γ [XMG1.2] 25 ug	Biolegend	505823
Anti‐NK‐1.1 Mouse Monoclonal Antibody (PE (Phycoerythrin)/Cy5) [clone: PK136], Size = 25 µg	Biolegend	108715
Anti‐CD80 Armenian Hamster Monoclonal Antibody (APC (Allophycocyanin)) [clone: 16‐10A1], Size = 25 µg	Biolegend	104713
FITC anti‐mouse CD86 [GL‐1] 50 µg	Biolegend	105005
PE anti‐mouse CD11c [N418] 50 µg	Biolegend	117307
Brilliant Violet 421 anti‐mouse CD3 [17A2] 125 µL	Biolegend	100227
Anti‐CD19 Rat Monoclonal Antibody (Brilliant Violet 421) [clone: 6D5], Size = 125 µL	Biolegend	115537
PE/Cyanine7 anti‐mouse CD86 Antibody	Biolegend	159207
Anti‐CD80 Armenian Hamster Monoclonal Antibody (APC (Allophycocyanin)) [clone: 16‐10A1], Size = 25 µg	Biolegend	104713
Brilliant Violet 711 anti‐mouse CD19 [6D5] 50 µg	Biolegend	115555
FITC anti‐mouse CD11c Antibody	Biolegend	117306
PerCP/Cyanine5.5 anti‐mouse CD45 Antibody	Biolegend	157207
Anti‐I‐A/I‐E Rat Monoclonal Antibody (Alexa Fluor 700) [clone: M5/114.15.2], Size = 25 µg	Biolegend	107621
UltraComp eBeads Plus Compensation Beads	Thermo Scientific	01‐3333‐42

### 7.8 Reagents for Flow Cytometry Analysis

Antibodies, viability dyes, and compensation beads used for flow cytometry are listed in Table 2.

### 7.9 Antigen Lymph Node Trafficking

#### 7.9.1 FITC‐Labeled Peptides

Mice were vaccinated subcutaneously at the tail base with 5 × 10^−8 ^mol of FITC‐SaPN‐OVA and 5 × 10^−8 ^mol of FITC‐OVA to each treatment group, respectively. After 6 h, the nearby inguinal lymph nodes were resected. Whole lymph nodes were imaged by the IVIS system to measure fluorescent signal intensity under the FITC channel.

#### 7.9.2 Cy3‐Labeled Peptides

Mice were vaccinated subcutaneously at the tail base with 5 × 10^−8 ^mol of Cy3‐SaPAC‐OVA, 5 × 10^−8 ^mol of Cy3‐OVA, 5 × 10^−8 ^mol of Cy3‐SaPN‐OVA along with 15 × 10^−8 ^mol of 1V209 and 5 × 10^−8 ^mol Cy3‐SaPN‐OVA to each treatment group respectively. Each injection bolus was separated into half doses on each side of the tail base. Then the axillary lymph nodes, both inguinal lymph nodes on either the left side or right side, the livers, the kidneys, the lungs, and the spleens were excised after 24 h. All tissue organs were imaged by the IVIS system. The fluorescent signal intensity of whole organs was measured under the Cy3 channel by the IVIS system.

#### 7.9.3 Lymph Node Dendritic Cells Staining and Flow Cytometry Analysis

Mice were vaccinated with peptides subcutaneously. After 24 h, mice were sacrificed, and inguinal lymph nodes were excised. Lymph nodes were ground on 70 µm cell strainers to collect single cells. LN cells were spun down and collected by centrifugation. LN cells were stained with an antibody mixture for 40 min on ice. After immunostaining, LN cells were resuspended in flow buffer and assessed by flow cytometry analysis.

### 7.10 Splenocytes Staining for Flow Cytometry Analysis and Inflammation Cytokine Profiling

Female mice (C57BL/6j) were vaccinated subcutaneously twice on day 0 and day 7 with 2.5 × 10^−8 ^mol of SaPAC‐OVA, 2.5 × 10^−8 ^mol of SaPN‐OVA, 2.5 × 10^−8 ^mol of SaPN‐OVA along with 7.5 × 10^−8 ^mol of 1V209 and 2.5 × 10^−8 ^mol of OVA epitope to each treatment group respectively. On day 9, mice were sacrificed, and spleens were dissected from mice. The splenocytes were collected by grinding spleens on 70 µm cell strainers. The splenocytes were spun down at 550 g for 5 min. The 1X Red Blood Cell Lysis buffer was used on splenocytes to remove red blood cells. The splenocytes were washed with PBS twice, then were resuspended in RPMI 1640 (with 10% FBS and 1% P/S). 500 000 splenocytes from each mouse were seeded onto every single well of IFNγ ELISpot plates (Mabtech) along with 40 ng of OVA epitope peptides as stimulus. After 24 h co‐incubation, both splenocytes and supernatant were collected. Splenocytes were digested and washed twice with PBS for immunostaining. After co‐culture with the antibody mixture for 40 min on ice. Splenocytes were washed with PBS twice and resuspended in flow buffer. Then splenocytes were assessed by flow cytometry. **Inflammation cytokine profiling**. The supernatant was collected from co‐culture media for each sample. 25 µL of supernatant was collected after 15 000 rcf centrifugation to remove cellular debris. Legendplex Mouse Inflammation Panel (13‐plex) with V‐bottom Plate (BioLegend, cat. # 740446) was used to measure the inflammatory cytokines’ concentration in supernatant by following the protocol. The data were analyzed at https://legendplex.qognit.com/.

### 7.11 ELISpot for Antigen Immunogenicity Determination

#### 7.11.1 SaPAC‐OVA Immunogenicity Test

Female mice (C57BL/6j) were vaccinated subcutaneously twice on day 0 and day 7 with 2.5 × 10^−8 ^mol of SaPAC‐OVA, 2.5 × 10^−8 ^mol of SaPN‐OVA, 2.5 × 10^−8 ^mol of SaPN‐OVA along with 7.5 × 10^−8 ^mol of 1V209 and 2.5 × 10^−8 ^mol of OVA epitope to each treatment group respectively. On day 9, mice were sacrificed, and spleens were dissected from mice. The splenocytes were collected by grinding spleens on 70 µm cell strainers. The splenocytes were spun down at 550 g for 5 min. The 1X Red Blood Cell Lysis buffer was used on splenocytes to remove red blood cells. The splenocytes were washed with PBS twice, then were resuspended in RPMI 1640 (with 10% FBS and 1% P/S). 500 000 splenocytes from each mouse were seeded onto every single well of IFNγ ELISpot plates (Mabtech) along with 40 ng of OVA epitope peptides as stimulus. After 24 h co‐incubation, the IFNγ plates were stained and visualized by following the protocol from Mabtech.

#### 7.11.2 4T1 Neoantigen Immunogenicity Test

Female mice (BALB/c) were vaccinated subcutaneously twice on day 0 and day 7 with 2.5 × 10^−8 ^mol of 4T1#2, 4T1#3, 4T1#5, 4T1#8, 4T1#10, 4T1#12, 4T1#13 and 4T1#14 respectively along with 200 µg Poly(I:C) as adjuvant to each treatment group. On day 9, mice were sacrificed, and spleens were dissected from mice. The splenocytes were collected by grinding spleens on 70 µm cell strainers. The splenocytes were spun down at 550 g for 5 min. The 1X Red Blood Cell Lysis buffer was used on splenocytes to remove red blood cells. The splenocytes were washed with PBS twice, then were resuspended in RPMI 1640 (with 10% FBS and 1% P/S). 500 000 splenocytes from each mouse were seeded onto every single well of IFNγ ELISpot plates (Mabtech) along with 40 ng of OVA epitope peptides as stimulus. After 24 h co‐incubation, the IFNγ plates were stained and visualized by following the protocol from Mabtech.

### 7.12 Pharmacokinetics Studies

FITC‐OVA and FITC‐SaPN‐OVA were synthesized by GL Biochem (Shanghai) Ltd. Mice were injected subcutaneously or intravenously with 2.5 × 10^−8^ mol FITC‐OVA or FITC‐SaPN‐OVA. Serum fluorescence was measured with a GE Amersham Typhoon 5 imager, and intravenous and subcutaneous curves were fitted with the equations shown in Figure S1E using SciPy in Python 3.8.

### 7.13 Lymph Node RNA Extraction

To extract total RNA from a lymph node using TRIzol (Thermo Fisher Scientific), the tissue sample is first homogenized in 1 mL of TRIzol reagent per 50–100 mg of tissue using a mechanical homogenizer until completely lysed. The homogenate is incubated at room temperature for 5 min to permit complete dissociation of nucleoprotein complexes. Subsequently, 0.2 mL of chloroform per 1 mL of TRIzol used is added, and the tube is shaken vigorously by hand for 15 s, followed by incubation at room temperature for 2–3 min. The sample is then centrifuged at 12 000 × g for 15 min at 4°C, which separates the mixture into an aqueous upper phase containing RNA, an interphase containing DNA, and a lower organic phase containing proteins and lipids. The aqueous phase is carefully transferred to a new tube without disturbing the interphase, and RNA is precipitated by adding 0.5 mL of isopropanol per 1 mL of TRIzol used. After incubation at room temperature for 10 min and centrifugation at 12 000 × g for 10 min at 4°C, the RNA pellet is washed with 75% ethanol, air‐dried, and resuspended in RNase‐free water. For small or fibrous lymph node samples, adding glycogen (5–10 µg) as a carrier during isopropanol precipitation can improve RNA yield and purity.

### 7.14 4T1(or MB49) Neoantigen Screening and Selection

Mouse blood, tumor cell lines, or tumor tissues were collected and sent to Novogene Technology Co., Ltd. for nucleic acid extraction, exome capture, and NovaSeq sequencing. Somatic mutations and transcriptome evidence were analyzed with VaxRank [39] and NetMHCpan [35,40]. The top 15 candidates were ranked by predicted binding and expression support; eight candidates were tested by IFN‐gamma ELISPOT after Poly(I:C)‐adjuvanted vaccination. The MB49 neoantigen was screened and selected with a similar workflow. A compact summary of mutation, mutant peptide, wild‐type counterpart, predicted MHC allele, and ELISPOT status is provided in Table S1.

### 7.15 Transcriptome Sequencing and Analysis

Tumor‐draining lymph node tissue was collected from mice after different types of conjugates were administered or mock‐treated. Samples after RNA extraction and passing RNA concentration quality control were then sent for transcriptome sequencing using the same platform and supplier as described above. To obtain clean sequence data, sequence adapters and low‐quality bases from raw data were trimmed using fastp (version 0.24.0) [41]. Mouse gene expression quantification was carried out with the help of Salmon (v1.10.0) [42] based on the clean data. Gene differential expression analysis was conducted by DESeq2 (v1.30.1) [43]; genes with adjusted *p*‐value below or equal to 0.05 and absolute fold changes between two conditions greater than or equal to 2 were considered significantly differentially expressed. Biology processes gene ontology analysis was conducted by clusterProfiler (v3.18.1) [44]. Immune cell functional enrichment score was calculated by xCell [45, 31] using a mouse‐human gene symbol transferred gene expression profile.

## Author Contributions

Y.W., Y.H., and J.‐D.H designed the study. Y.W. performed major experiments. J.H. performed the in silico analysis. Y.W., J.H., and J.‐D.H. analysed the data. W.L. and R.L. performed animal studies. Y.L. assisted in characterization experiments. Y.W., J.H., Y.H., C.C., and J.‐D.H. wrote the manuscript. Y.‐F.H., J.‐H.M., B.‐Z.Z., H.C., S‐F.C. and J.‐D.H. revised the manuscript. Generative AI Gemini 3.1, Grok 4, and DeepSeek V2 were used in writing, schematic diagrams, and analysis.

## Funding

The research was supported by the National Key Research and Development Program of China (2021YFA0910700), Shenzhen Medical Research Fund (D2402020), a Special Research Fund from HKU, Shenzhen Key Laboratory for Cancer Metastasis and Personalized Therapy (ZDSYS20210623091811035), Seed Fund for Translational and Applied Research (202111160012), the Innovation and Technology Support Programme (Seed Project; ITS/365/23), and National Natural Science Foundation of China (Major Program; 92369201).

## Conflicts of Interest

Y.‐F.H. is currently the CEO of BayVax Biotech Limited. J.‐D.H. and Y.‐F.H. are named inventors on a patent application related to this work, assigned to Versitech Limited and published as U.S. Patent Application Publication No. US 2025/0049902 A1 (U.S. Application No. 18/721,343), entitled “Method of Developing a Peptide‐Based Vaccine Conjugated with 1V209.”

## Supporting information




**Supporting File**: advs77632‐sup‐0001‐SuppMat.docx.

## Data Availability

The raw sequencing datasets we generated (Bulk RNA‐seq and whole exome sequencing) have been deposited in the NCBI BioProject: PRJNA1519071.
