# Supplementary material for: Self‐Assembling Peptide‐Adjuvant Conjugate (SaPAC) Platform for Precision Cancer Immunotherapy
Source: Adv Sci (Weinh). 2026 Oct 2:e77632. Online ahead of print. doi: 10.1002/advs.77632 (PMC13633104; doi:10.1002/advs.77632)
Supplement: Supplementary file 1 [file ADVS-9999-e77632-s001.docx]

**Supporting information**

**Table S1. Compact neoantigen summary for MB49 and 4T1 vaccine constructs.**

| **ID** | **Tumor Model** | **Genomic Position** | **Gene** | **RNA reads supporting variant allele** | **RNA reads supporting reference allele** | **AA change** | **Sequence** | **Epitope sequence** | **MHC Allele** | **WT sequence** | **ELISPOT validation status** |
| --- | --- | --- | --- | --- | --- | --- | --- | --- | --- | --- | --- |
| 4T1#1 | 4T1 | chr18:g.31827321C>T | Wdr33 | 43 | 66 | p.H13Y | MATEIGSPPRFF_Y_MPRFQHQAPRQL | GSPPRFFYM | H-2-Dd | GSPPRFFHM | n.s. |
| 4T1#2 | 4T1 | chrX:g.85711101T>C | Gk | 34 | 42 | p.K511R | AEESEIRYSTWK_R_AVMKSIGWVTTQ | RYSTWKRAV | H-2-Kd | RYSTWKKAV | Significant (p<0.0001) |
| 4T1#3 | 4T1 | chr5:g.151045164C>G | Stard13 | 3 | 22 | p.Q1017H | FVQWKVVERLDKQTEIY_H_YVLNSMV | IYHYVLNSM | H-2-Kd | IYQYVLNSM | Significant (p<0.0001) |
| 4T1#4 | 4T1 | chr10:g.119213632T>G | Cand1 | 21 | 109 | p.K403T | REENVKADVFHAYLSLL_T_QTCPVQS | AYLSLLTQT | H-2-Kd | AYLSLLKQT | n.s. |
| 4T1#5 | 4T1 | chr5:g.36824144C>T | Man2b2 | 17 | 70 | p.V210I | SPSLSGQQEIFTH_I_MDHYSYCTPSH | GQQEIFTHI | H-2-Kd | GQQEIFTHV | Significant (p<0.0001) |
| 4T1#6 | 4T1 | chr17:g.43453098G>A | Adgrf5 | 3 | 136 | p.V1304M | GSSTP_M_FSMSSPISRRFNNLFGK | PMFSMSSPI | H-2-Kd | PVFSMSSPI | n.s. |
| 4T1#7 | 4T1 | chr17:g.56211643C>T | Dpp9 | 11 | 61 | p.D54N | RKSSGLIVSKAPH_N_FQFVQKPDESG | KAPHNFQFV | H-2-Dd | KAPHDFQFV | n.s. |
| 4T1#8 | 4T1 | chr16:g.94451047C>T | Ttc3 | 8 | 89 | p.L1533F | TEENKISKTELDWF_F_QDLDREIKKW | FFQDLDREI | H-2-Kd | FLQDLDREI | Significant (p=0.0005) |
| 4T1#9 | 4T1 | chr11:g.100695233C>A | Dhx58 | 66 | 39 | p.R654L | LETPRGKIQAKKWS_L_VPFSIPVFDI | KWSLVPFSI | H-2-Kd | KWSRVPFSI | Significant (p=0.0001) |
| 4T1#10 | 4T1 | chr7:g.24472004G>A | Plaur | 28 | 357 | p.R140H | RGREQSLQC_H_YPTEHCIEVVTLQST | CHYPTEHCI | H-2-Kd | CRYPTEHCI | Significant (p<0.0001) |
| 4T1#11 | 4T1 | chr6:g.135078913C>A | Gprc5a | 53 | 56 | p.F119L | FSCLLAHA_L_NLIKLVRGRKPLSWLV | AHALNLIKL | H-2-Kd | AHAFNLIKL | Significant (p=0.0002) |
| 4T1#12 | 4T1 | chr19:g.47898131G>C | Itprip | 43 | 48 | p.A15G | MELFRVCLVVVT_G_IINHPLLFPREN | TGIINHPLL | H-2-Dd | TAIINHPLL | Significant (p<0.0001) |
| 4T1#13 | 4T1 | chr15:g.12150180A>C | Zfr | 43 | 84 | p.K411T | ADAYAAHIRGAKHQKVV_T_LHTKLGK | KVVTLHTKL | H-2-Kd | KVVKLHTKL | Significant (p<0.0001) |
| 4T1#14 | 4T1 | chr7:g.44535941G>C | Pold1 | 10 | 80 | p.A745G | VESKYTVENGYD_G_NAKVVYGDTDSV | GYDGNAKVV | H-2-Kd | GYDANAKVV | Significant (p<0.0001) |
| MB49 neoantigen | MB49 | chr8:g.116993366T>C | Gcsh | 154 | 0 | p.T21A | SLRVVAYSLRTAL_A_FCSPRPCVPSAAA | YSLRTALAF | H-2-Kb | YSLRTALTF | Significant (p<0.0001) |

**Table S2. Consolidated physicochemical characterization of SaPAC/SaPN nanoparticles.**

| **Construct** | **Peptide concentration** | **Buffer/condition** | **Method** | **Median of hydrodynamic diameter ( +/- sem)** | **Zeta potential (+/- sem)** | **TEM morphology** | **Independent batches** |
| --- | --- | --- | --- | --- | --- | --- | --- |
| SaPN-OVA | 30 μg/mL | Milli Q water | TEM; NTA/ZetaView | 135 +/- 7 nm | 23.59 +/- 0.48 mV | Nanoparticles | 5 |
| SaPAC-OVA | 5 μg/mL | Milli Q water | TEM; NTA/ZetaView | 169 +/- 18 nm | 28.92 +/- 0.89 mV | Nanoparticles | 5 |
| SaPAC(ii)-OVA | 5 μg/mL | Milli Q water | TEM; NTA/ZetaView | 125 +/- 4 nm | 29.89 +/- 1.64 mV | Nanoparticles | 5 |
| SaPAC(iii)-OVA | 5 μg/mL | Milli Q water | TEM; NTA/ZetaView | 133 +/- 3 nm | 30.71 +/- 1.25 mV | Nanoparticles | 5 |
| SaPAC-MB49 | 30 μg/mL | Milli Q water | TEM; NTA/ZetaView | 169 +/- 4 nm | 14.56 +/- 0.70 mV | Nanoparticles | 3 |
| SaPAC-4T1#2 | 10 μg/mL | Milli Q water | TEM; NTA/ZetaView | 125 +/- 16 nm | 37.66 +/- 0.22 mV | Nanoparticles | 3 |
| SaPAC-4T1#3 | 30 μg/mL | Milli Q water | TEM; NTA/ZetaView | 144 +/- 4 nm | 32.28 +/- 0.86 mV | Nanoparticles | 3 |
| SaPAC-4T1#13 | 30 μg/mL | Milli Q water | TEM; NTA/ZetaView | 116 +/- 2 nm | 31.56 +/- 0.47 mV | Nanoparticles | 3 |

**Figure S1. The SaPN platform enhances antigen depot effect and pharmacokinetic exposure.** (A,B) Pharmacokinetic model describing absorption and clearance rates that influence peptide antigen bioavailability in lymphoid organs. (C,D) FITC-SaPN-OVA and FITC-OVA serum fluorescence after subcutaneous or intravenous injection. (E) Fitting equations and representative fitted curves generated using SciPy in Python 3.8. (F,G) Calculated clearance and absorption rates.

**Figure S2. SaPN enhances peptide antigen depot retention and lymphoid-organ bioavailability.** (A) Whole-body IVIS fluorescence 6 h after subcutaneous injection. (B) IVIS fluorescence of excised inguinal LNs 6 h post-injection. (C) EC50 fluorescence calibration. (D) Serum fluorescence over 24 h after subcutaneous injection. (E) Quantification of inguinal draining LN fluorescence. Significance was assessed by unpaired two-tailed Student's t-test.

**Figure S3. Site-selective synthesis and construct map of SaPAC-OVA variants.** (A) The protected peptide precursor (Compound 1) on Rink amide resin contains Boc-protected non-target lysines and Dde-protected target lysines. Selective Dde removal with 2% hydrazine in N,N-dimethylformamide exposes the designated lysine epsilon-amines for coupling of carboxylic-acid-functionalized 1V209. Global Fmoc/Boc deprotection and resin cleavage yield the final SaPAC-OVA conjugate. (B) Construct map of SaPN-OVA, SaPAC-OVA, SaPAC(ii)-OVA, and SaPAC(iii)-OVA. The map identifies the cationic region, enzyme-cleavable linker, OVA epitope, and lysine residues conjugated to 1V209 (K*). A, alanine spacer; K*, lysine side chain covalently conjugated to 1V209.

**Figure S4. Physicochemical characterization of SaPAC-OVA variants.** (A) Reversed-phase HPLC profiles of SaPAC-OVA, SaPAC(ii)-OVA, and SaPAC(iii)-OVA. (B) High-resolution mass spectra confirming SaPAC-OVA, SaPAC(ii)-OVA, and SaPAC(iii)-OVA. (C,D) Representative NTA size distributions of SaPN-OVA and SaPAC-OVA, respectively. (E) ZetaView/NTA hydrodynamic diameter and zeta-potential measurements for peptide constructs and vaccine nanoparticles. Full numerical size, zeta-potential, buffer-condition, and independent-batch data are summarized in Table S2.

**Figure S5 Normalization of Cy3 signal to concentration of Cy3-SaPAC-OVA, Cy3-SaPN-OVA and Cy3-OVA.** Normalized percent fluorescence of Cy3-OVA, Cy3-SaPN-OVA and Cy3-SaPAC-OVA in different concentrations in 1xPBS measured by biofluorescent IVIS. The data were fitted by a solid line using SciPy in Python 3.8 **A)** Calculated effective concentration with half of maximum fluorescence (EC50) for different peptide or peptide-adjuvant conjugates, Cy3-OVA, Cy3-SaPN-OVA and Cy3-SaPAC-OVA respectively. **B)** EC50 for each peptide or vaccine were compared in a bar graph.

**Figure S6. Biodistribution of different vaccine formulations 24 h after subcutaneous vaccination.** Molar-equivalent Cy3-OVA, Cy3-SaPN-OVA, Cy3-SaPN-OVA + 1V209, or Cy3-SaPAC-OVA were injected into mice (n = 4/group). SaPAC-OVA contains three covalently linked 1V209 moieties; the Cy3-SaPN-OVA + 1V209 group received three molar equivalents of unconjugated 1V209. IVIS fluorescence was measured in (A) liver, (B) lung, (C) kidney, (D) spleen, and (E) distal lymph nodes.

**Figure S7.** **DC2.4 cell line presentation after 24 h co-culture with various vaccines or peptides**

**Figure S8. Cytokine profiling of supernatants from vaccinated mouse splenocytes restimulated with OVA.** TNF-alpha concentration was increased in the SaPAC group (P = 0.0251 versus WT).

**Figure S9. Transcriptomic analysis of innate immune activation in MB49 tumor-draining lymph nodes after SaPAC-MB49 treatment.** (A) Treatment timeline. (B) Heatmap of phagocytosis-related genes. (C) Venn diagram of significantly upregulated genes. (D) GO enrichment analysis. (E) PD-1 expression. (F,G) ssGSEA scores for plasmacytoid dendritic cells and macrophages. Data are mean +/- SEM; significance was assessed by unpaired two-tailed Student's t-test.

**Figure S10. Individual B16-OVA tumor growth curves and endpoint tumors from Figure 5A-F.** (G) Red crosses indicate mice that died before endpoint.

**Figure S11** **Characterization for SaPAC-MB49.** A) Mass spectrometry for SaPAC-MB49. B) High-resolution liquid chromatography for SaPAC-MB49.

**Figure S12. Individual MB49 tumor growth curves from Figure 5G-I.**

**Figure S13. 4T1 neoantigen screening and selection.** (A) Neoantigen screening pipeline using sequencing, somatic mutation calling, NetMHCpan ranking, and peptide synthesis. (B) IFN-gamma ELISPOT quantification after candidate peptide vaccination with Poly(I:C). (C) Selected neoantigens 4T1#2, 4T1#3, and 4T1#13 with mutation positions and sequences. (D) Wild-type counterpart peptide vaccination showing no significant cross-reactive immunogenicity. Statistical significance was assessed by an unpaired two-tailed Student's t-test.

**Figure S14. Therapeutic evaluation of triSaPAC-4T1 monotherapy in the orthotopic 4T1 model.** Treatment timeline and vaccination schematic for 4T1 neoantigen mixture, triSaPAC-4T1, 4T1 neoantigen + Poly(I:C), and 4T1 neoantigen + 1V209. Tumor volumes were recorded every 2-3 days until day 16. Significance was assessed by unpaired two-tailed Student's t-test.
